# SARS-CoV-2 accessory proteins reveal distinct serological signatures in children

**DOI:** 10.1038/s41467-022-30699-5

**Published:** 2022-05-26

**Authors:** Asmaa Hachim, Haogao Gu, Otared Kavian, Masashi Mori, Mike Y. W. Kwan, Wai Hung Chan, Yat Sun Yau, Susan S. Chiu, Owen T. Y. Tsang, David S. C. Hui, Chris K. P. Mok, Fionn N. L. Ma, Eric H. Y. Lau, Gaya K. Amarasinghe, Abraham J. Qavi, Samuel M. S. Cheng, Leo L. M. Poon, J. S. Malik Peiris, Sophie A. Valkenburg, Niloufar Kavian

**Affiliations:** 1 HKU-Pasteur Research Pole, School of Public Health, Li Ka Shing Faculty of Medicine, The University of Hong Kong, Hong Kong SAR, China; 2 Division of Public Health Laboratory Sciences, School of Public Health, Li Ka Shing Faculty of Medicine, The University of Hong Kong, Hong Kong SAR, China; 3 Department of Mathematics, Université de Versailles Saint-Quentin, Versailles, France; 4 Research Institute for Bioresources and Biotechnology, Ishikawa Prefectural University, Nonoichi, Ishikawa Japan; 5 Department of Pediatric and Adolescent Medicine, Princess Margaret Hospital, Hospital Authority of Hong Kong, Hong Kong SAR, China; 6 Department of Pediatrics, Queen Elizabeth Hospital, Hospital Authority of Hong Kong, Hong Kong SAR, China; 7 Department of Pediatric and Adolescent Medicine, The University of Hong Kong and Queen Mary Hospital, Hospital Authority of Hong Kong, Hong Kong SAR, China; 8 Infectious Diseases Centre, Princess Margaret Hospital, Hospital Authority of Hong Kong, Hong Kong SAR, China; 9 Department of Medicine and Therapeutics, Prince of Wales Hospital, The Chinese University of Hong Kong, Hong Kong SAR, China; 10 The Jockey Club School of Public Health and Primary Care, The Chinese University of Hong Kong, Hong Kong SAR, China; 11 WHO Collaborating Centre for Infectious Disease Epidemiology and Control, School of Public Health, Li Ka Shing Faculty of Medicine, The University of Hong Kong, Hong Kong SAR, China; 12 Department of Pathology and Immunology, Washington University School of Medicine in St. Louis, St. Louis, MO USA; 13 Doherty Institute of Infection and Immunity, Department of Microbiology and Immunology, The University of Melbourne, Melbourne, Australia; 14 Faculté de Médecine Université Paris Descartes, Sorbonne Paris Cité, Assistance Publique–Hôpitaux de Paris, Hôpital Universitaire Paris Centre, Centre Hospitalier Universitaire Cochin, Service d’Immunologie Biologique, Paris, France; 15 Institut Cochin, INSERM U1016, Université Paris Descartes, Sorbonne Paris Cité, Paris, France

**Keywords:** Infectious diseases, Adaptive immunity

## Abstract

The antibody response magnitude and kinetics may impact clinical severity, serological diagnosis and long-term protection of COVID-19, which may play a role in why children experience lower morbidity. We therefore tested samples from 122 children in Hong Kong with symptomatic (*n* = 78) and asymptomatic (*n* = 44) SARS-CoV-2 infections up to 200 days post infection, relative to 71 infected adults (symptomatic *n* = 61, and asymptomatic *n* = 10), and negative controls (*n* = 48). We assessed serum IgG antibodies to a 14-wide antigen panel of structural and accessory proteins by Luciferase Immuno-Precipitation System (LIPS) assay and circulating cytokines. Infected children have lower levels of Spike, Membrane, ORF3a, ORF7a, ORF7b antibodies, comparable ORF8 and elevated E-specific antibodies than adults. Combination of two unique antibody targets, ORF3d and ORF8, can accurately discriminate SARS-CoV-2 infection in children. Principal component analysis reveals distinct pediatric serological signatures, and the highest contribution to variance from adults are antibody responses to non-structural proteins ORF3d, NSP1, ORF3a and ORF8. From a diverse panel of cytokines that can modulate immune priming and relative inflammation, IL-8, MCP-1 and IL-6 correlate with the magnitude of pediatric antibody specificity and severity. Antibodies to SARS-CoV-2 internal proteins may become an important sero surveillance tool of infection with the roll-out of vaccines in the pediatric population.

## Abstract (web summary)

The antibody response of children to SARS-CoV-2 is less well studied than in adults. Here Hachim et al. show that children have reduced antibody levels to structural proteins and suggest that the predominance of antibody responses to non-structural proteins can be used to discriminate infection and vaccination.

## Introduction

The spectrum of SARS-CoV-2 infection ranges from asymptomatic to lethal infection, with the immune response playing a major role in the pathogenicity and outcome of COVID-19^1^. Children are generally less affected clinically by SARS-CoV-2 infection and the morbidity and mortality observed in adults increases progressively with age. The viral loads in the upper respiratory tract are reportedly comparable between children of all ages and adults^2^. Various immune functions and physiological differences have also been implicated in differential outcomes with age, such as lower ACE2 expression in children^3^, pre-existing immunity to common cold coronaviruses (CCoV)^4^, elevated baseline IgM^5^, immuno-senescence, inflammatory state^6^, innate immune responses^7^, auto-antibodies^8^, and off-target “trained immunity”^9,10^. Multisystem inflammatory syndrome (MIS-C) that can develop in children after infection with SARS-CoV-2 is a rare exception (0.002% of pediatric cases) to the generally milder clinical disease observed^11^.

Serology is crucial for determining infection attack rates in the population and for assessing the response to current vaccines to curb the global pandemic. Large epidemiological studies reported that children only represent 1–2% of all SARS-CoV-2 cases in 2020^12,13^. Most serological tests available rely either on neutralizing antibodies or on the detection of binding antibodies targeting the Spike (S) or the Nucleocapsid (N) proteins of the virus^14^. Across three commercial diagnostics S-based assays, children show a lower rate of seroconversion than adults despite having PCR confirmed infection and comparable viral loads^15^, therefore S-based serology in children may not be an accurate marker of recent infection and underestimate seroprevalence in children. Furthermore, N-specific antibody waning is more pronounced than S^16^ and children are more likely to test S-antibody positive even in the absence of vaccination^17^. Therefore, N-based serology in children is also limited^18^ compared to adults^19^. Saliva-based approaches may offer an easier sampling site with long-term duration of IgG and N-specific responses^20,21^ over S^22^ and IgA, even in children^23^.

We have previously demonstrated that antibodies that are directed against non-structural proteins of the virus, namely ORF3d and ORF8, can be used for accurate diagnosis of SARS-CoV-2 infection in adults^24^. Further studies have also contributed data on the SARS-CoV-2 antibody responses to the virus accessory proteins in the adult population^24–26^ but these accessory specific antibody data are lacking for children. For instance, ORF3d, ORF6, and ORF7a, which have been reported to be potent interferon antagonists that may play a role in immune evasion^27–29^, or ORF8, which seems to participate to the downregulation of MHC I molecules and to viral pathogenesis^30,31^. In addition, an imbalanced production of cytokines is responsible of severe COVID-19 outcomes in adults^32^ which may modulate seropositivity. Finely tuned and balanced antibody response in relation to  cytokine responses may impact SARS-CoV-2 infection outcomes, thus the breadth and magnitude of the specificity of antibody responses to non-structural proteins may indicate the extent of virus replication and thus immune control.

In the present study, children and adults with SARS-CoV-2 RT-PCR confirmed infection were used to determine the antibody specificity to a comprehensive panel of 14 different structural and accessory proteins by Luciferase Immuno-Precipitation System (LIPS) (l). Antibody responses were then compared relative to circulating levels of a selected panel of cytokines, known to modulate the antibody response and inflammation. The majority of samples were collected between April to November 2020, before the roll-out of COVID-19 vaccines. Furthermore, due to intensive contact tracing and case-finding measures in Hong Kong, asymptomatic pediatric cases with RT-PCR confirmed infections have been included, which represents a rare entity in most countries and are a unique aspect of our study.

## Results

### Different levels of antibodies to structural proteins in children and adults with SARS-CoV-2 infection

We used the unbiased and quantitative LIPS platform to determine the antibody responses to an extensive panel of 14 antigens from structural and non-structural SARS-CoV-2 proteins in plasma samples from a cohort of infected children, in comparison to adults and controls in Hong Kong and the USA (Table 1).

**Table 1:** Subject cohorts details.

			Pediatric COVID-19		Adult COVID-19		Negative	
			*N* (%)		*N* (%)		*N* (%)	
Patients	Samples		254		71		48	
	Individuals		122		71		48	
Symptoms	Asymptomatic		44 (36%)		10 (14%)		–	
	Mild		78 (64%)		50 (70.5%)		–	
	Severe		0		11 (15.5%)			
Age			Mean±stdev	10.8 ± 4.9 years	Mean ± stdev	43.7 ± 19.3 years	Mean ± stdev	–
			0–8 years	45 (37%)	Median	45	Adults (*N* = 28)	18–65
			8–12 years	33 (27%)	Min	18	Pediatrics (*N* = 20)	11–18
			12–18 years	44 (36%)	Max	84		
Sex	Female		49 (40%)		34 (48%)		–	
	Male		73 (60%)		37 (52%)		–	

**Fig. 1:**
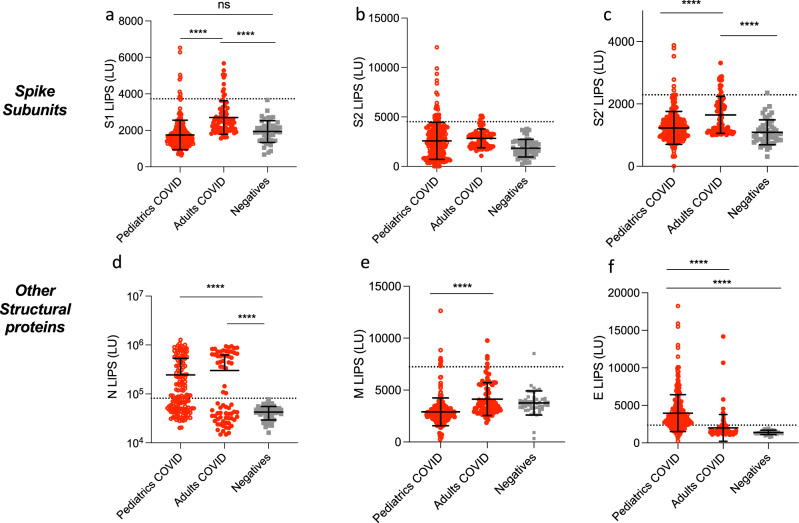
Comparison of antibody responses to SARS-CoV-2 structural proteins in children and in adults with COVID-19. Antibodies against the SARS-CoV-2 structural proteins Spike S1 subunit (S1) (**a**), Spike S2 subunit (**b**), Spike S2′ subunit (**c**), Nucleocapsid (N) (**d**), Membrane (M) (**e**), and Envelope (E) (**f**) were measured by LIPS from samples from pediatrics COVID-19 (*n* = 254) or adult patients (*n* = 71), and negative controls (*n* = 48). Background no plasma values were subtracted. Experiments were repeated twice. The cutoff value is shown by the dotted line and was based on the mean plus 3 × s.d. of the negative control group. All data represent individual time point IgG responses and to a lesser extent IgA and IgM based on protein A/G bead binding by LIPS and the mean ± stdev. Two-sided *P* values were calculated using the Mann–Whitney U test. * shows statistical significance between COVID-19 patients versus negative controls. **p < 0.01, ***p < 0.001, ****p < 0.0001. Significant *p* values for pediatrics versus adults are S1 (**a**) *p* < 0.0001, S2′ (**c**) *p* < 0.0001, M (**e**) *p* < 0.0001, E (**f**) *p* < 0.0001. Significant *p* values for pediatrics versus negatives are N (**d**) *p* < 0.0001, M (**e**) *p* = 0.0008, E (**f**) *p* < 0.0001.

Our first dataset represents the total cohort of SARS-CoV-2 infected cases of mixed time points and symptoms to determine the overall antibody specificity in children (mean ± stdev: 39 ± 47 days, range: 0–206 days), adults (mean ± stdev: 20 ± 23 days, range: 0-123 days) and negative controls (Table 1: adults with asymptomatic, mild, or severe disease, children with asymptomatic or mild disease, and negative controls). S and N antibodies are the most widely used antibodies in COVID-19 serology testing worldwide. We therefore first determined the levels of antibodies to different S sub-units by using 3 different S constructs in the LIPS assay: S1 which contains the RBD domain, S2, and the S2′ cleaved subunit (Fig. 1). The levels of the two Spike antibodies, S1 and S2′ were markedly lower in children compared to the adult cohort (both *p* < 0.0001, Fig. 1a, c), whereas no difference was observed for S2 antibodies, revealing different antigenicity for the two Spike isoforms S2 and S2′ (Fig. 1b)^33^. Moreover, N antibodies were significantly elevated in the pediatric COVID-19 cohort relative to negative controls (2.45 × 10^5^ ± 2.8 × 10^5 ^LU versus 4.15 × 10^4^ ± 1.5 × 10^5 ^LU (*p* = 0.0045), but did not differ from levels observed in adults (Fig. 1d).

We also assessed by LIPS antibodies to other structural proteins Matrix (M) and Envelope (E), which are not widely measured in serology. As for S1 and S2′, we found that M antibody levels were lower in infected children compared to infected adults (*p* < 0.0001, Fig. 1e), but were still significantly higher than negative controls. E antibodies had an opposite effect, and were significantly elevated in the pediatric COVID-19 cohort (Fig. 1f) compared to both adult COVID-19 (*p* = 0.0006) and negative controls (*p* < 0.0001). This antigen panel revealed that N and E were the best-performing antigens for diagnostics (based on a cut-off of the negative mean + 3x standard deviations) in the pediatrics population with 65% sensitivity and 100% specificity for N and 78% sensitivity and 100% specificity for E (Fig. 1d, f), which contrasts to our previous analysis in an adult population where E was not immunogenic^24^.

### Increased antibody response to the accessory protein ORF8 in the pediatric SARS-CoV-2 infected population

**Fig. 2:**
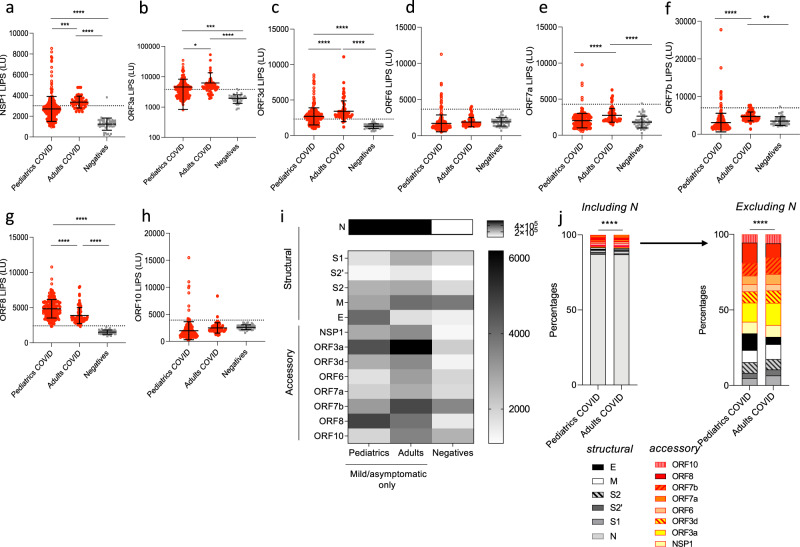
Diverse distribution of antibody responses to single SARS-CoV-2 proteins in children and adults with asymptomatic/mild COVID-19. **a**–**h** Antibody levels to accessory proteins of SARS-CoV-2. Antibodies against NSP1 (**a**) (in ORF1ab), and other ORFs (ORF3a (**b**), ORF3d (**c**), ORF6 (**d**), ORF7a (**e**), ORF7b (**f**), ORF8 (**g**), and ORF10 (**h**)) were measured in pediatric (*n* = 254) and adult (*n* = 71) COVID-19 cases and negative controls (*n* = 48) by LIPS to cover all the ORFs of the virus. Significant *p* values for pediatrics versus adults are NSP1 (**a**) *p* = 0.0005, ORF3d (**b**) *p* < 0.0001, ORF7a *p* < 0.0001, ORF7b *p* < 0.0001, ORF8 *p* < 0.0001. Significant *p* values for pediatrics versus negatives are NSP1 (**a**) *p* < 0.0001, ORF3d (**b**) *p* < 0.0001, ORF7a *p* < 0.0001, ORF8 *p* < 0.0001, ORF10 *p* < 0.025. **i**, **j** Comparison of the antibody distribution in asymptomatic/mild children and adults. The cutoff value is shown by the dotted line and was based on the mean plus 3 × s.d. of the negative control group. **i** Heatmap comparing the mean concentrations (LU) for structural (N, S, S1, S2′, S2, M, E) and accessory proteins (NSP1, ORF3a, ORF3d, ORF6, ORF7a, ORF7b, ORF10) responses in the COVID-19 pediatric, asymptomatic/mild adult populations (excluding severe cases) and negative controls. **j** Percentages of single antibody levels to SARS-CoV-2 antigens of the cumulative SARS-CoV-2 antibody response in COVID-19 children and asymptomatic/mild adults (excluding severe cases) for the antigen panel including and excluding N. The asymptomatic/mild pediatric and adult distributions were compared using a Chi-square test for expected versus observed distributions. Expected (adult) and observed (pediatric) percentages are detailed in Supplementary Table 3. *P* < 0.0001 for pediatric (observed) versus adult (expected) distribution of both antigen panels including N (left) and excluding N (right). Experiments were repeated twice. Two-sided *P* values were calculated using the Mann–Whitney U test. * shows statistical significance between COVID-19 patients versus negative controls. *p < 0.05, **p < 0.01, ***p < 0.005, ****p < 0.0001. Data in (**a**–**h**) represents individual time point LIPS responses and the mean ± stdev, data in (**i**) represents mean values (LU), data in (**j**) represents percentages.

We next investigated the levels of antibodies directed against the non-structural protein 1 (NSP1) and all the ORF proteins of the virus. In line with our previous study^24^, infected adults had elevated levels of NSP1, ORF3a, ORF3d, ORF7a, ORF7b, and ORF8 antibodies compared to negative controls (*p* < 0.0001, *p* < 0.0001, *p* < 0.0001, *p* = 0.05, *p* = 0.0009, *p* < 0.0001, Fig. 2a–c and e–g). No detectable levels of ORF6 and ORF10 antibodies were found in infected adults (*p* = 0.8691 and *p* = 0.999, respectively, Fig. 2d, h). We observed that the COVID-19 children cohort displayed significantly lower levels of ORF3a, ORF7a, ORF7b antibodies than the COVID-19 adult cohort (*p* = 0.0001, *p* < 0.0001 and *p* < 0.0001, respectively, Fig. 2b, e, f). The magnitude of antibody responses to NSP1 and ORF3d (previously referred to as ORF3b^24^, as ORF3d is within frame of ORF3b but ORF3b is not expressed^34^) were comparable in the pediatric COVID-19 and adult COVID-19 populations (Fig. 2a, c).

ORF8 antibody levels were found significantly elevated in pediatric COVID-19 samples compared to adults (*p* < 0.0001, Fig. 2g). In terms of performance as a diagnostic test, ORF8 antibodies by LIPS allows for the detection of nearly all the pediatric population with a sensitivity of 99.2% and specificity of 100% (Fig. 2g). These results were then confirmed in an in-house ELISA assay for IgG binding using recombinant proteins in both adult and pediatric plasma samples, where ORF8-protein^35^ binding antibodies showed 79% sensitivity and 98.4% specificity (Supplementary Fig. 1c), whereas N and Spike-protein binding antibodies showed a sensitivity of 88% and 11% and a specificity of 97% and 99%, respectively (Supplementary Fig. 1d, e). Furthermore, ORF8 remains a specific diagnostic tool for SARS-CoV-2 infection in vaccinated conditions. ORF8 specific IgG was assessed following vaccination with whole inactivated virion Coronavac and Spike mRNA lipoprotein BNT162b2 (Supplementary Fig. 1f), showing ORF8 is likely not incorporated within the virion.

We then compared the cumulative SARS-CoV-2 antibody responses from asymptomatic/mild only COVID-19 children and adult in a heatmap (Fig. 2i) and as percentages of the total SARS-CoV-2 structural and accessory antibody response (Fig. 2j). Due to the immunodominant effect of the N protein, anti-N antibodies substantially dominate the SARS-CoV-2 humoral response detected by LIPS in both populations (Fig. 2i), which is consistent with our previous findings in the adult population^24^. There was a significant difference in the distribution of the overall specificity of pediatrics and asymptomatic/mild COVID-19 adults (*p* < 0.0001 for “observed” pediatric distribution compared with “expected” adult distribution, Fig. 2j, Supplementary Fig. 1a, Supplementary Table 3). In the adult population antibody levels of S1, M, ORF3a and ORF7b represented a higher percentage of the response in adults than children (Fig. 2j and Supplementary Fig. 1a). While, ORF8 and E antibody responses represented a higher percentage in the pediatric population with 13.4% versus 9.2% and 11% versus 4.8%, respectively (Fig. 2j and Supplementary Fig. 1a). The remaining antigens, S2, S2′, N, NSP1, ORF3d, ORF6, ORF7a, ORF10, represent a similar percentage in both populations and hence are not contributing to the differences observed in specificity. Furthermore, there are no differences in total IgG serum concentration with age or infection, despite differences in the magnitude and specificity of SARS-CoV-2 antibodies in adults versus children (Supplementary Fig. 1b).

### SARS-CoV-2 antibody specificity using clusters of points and principal component analysis

**Fig. 3:**
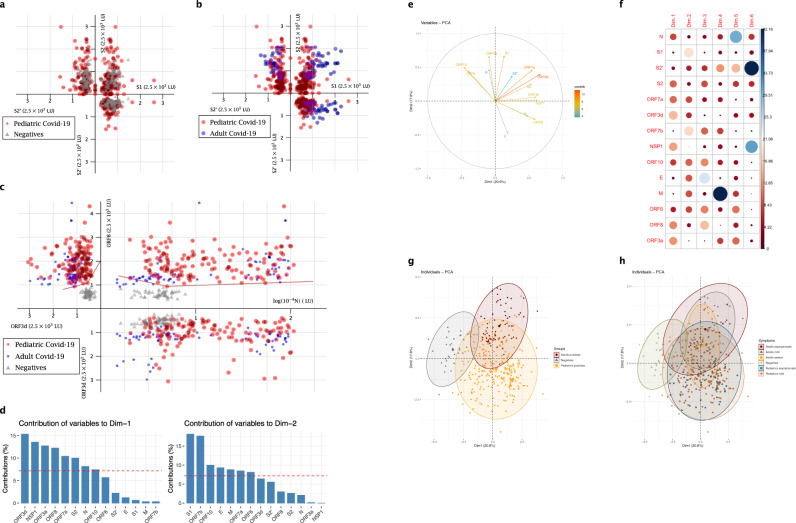
Representation of the pediatric COVID-19 population as a cluster of points for relevant antibody combinations and principal component analysis (PCA). **a**, **b** Cluster representation of S1, S2′, S2 antibodies combination. **a** The pediatric COVID-19 population versus the negative population, **b** the pediatric COVID-19 population versus the adult COVID-19 population (for the sake of clarity, the three populations were not represented on the same graph here). **c** Cluster representation of N, ORF3d, ORF8 antibodies combination, for the pediatric COVID-19 population versus the adult COVID-19 population and the negative population. Samples are represented according to their values of SARS-CoV-2 individual LIPS antibodies as (x, y, z) in the space. For the sake of clarity, only *n* = 144 pediatric COVID-19 (red), *n* = 71 adult COVID-19 (blue), and *n* = 48 negative (gray) samples are represented (**a**–**c**). **d**–**f** PCA of 14 antibodies analyzed in COVID-19 pediatric patients. Dim1 explains 20.6% of the variation, while Dim2 explains 17.7% of the variation. **d** Contribution of variables on dimensions 1 and 2. The red dashed line on the graph above indicates the expected average contribution. **e** Correlation circle and contributions. The scale of contributions is indicated (right). **f** Summary of contribution of variables on the different dimensions. **g**, **h** Factorial plots of PCA on dimensions 1 and 2. The plot is colored by sample types (Adults positives, Pediatrics positives, negative controls) (**g**), the largest point in shape in each group is the group mean point (circle is for Adult positives, squares for Pediatric positives, triangle for Negative controls) or by symptoms (**h**).

A cluster of points depicts each individual sample in a more complete way than a classical single statistical comparison, as it considers a combination of three (or more) different parameters taken together and the relevant relations of these parameters. To decipher the SARS-CoV-2 antibody specificity in children, we used relevant antibody combinations to represent the COVID-19 pediatric samples in clusters of points relative to negative controls and COVID-19 adult populations (Fig. 3a–c and Supplementary Fig. 2).

First, the cluster representing the three antibodies to the S subunit antigens S1, S2′, S2 confirmed that the pediatric population has a S antibody profile that is more closely comparable to negative controls (Fig. 3a) than an adult COVID-19 response by LIPS (Fig. 3b). Further cluster analysis of antibodies to other structural proteins N, M, and E reveals that the COVID-19 children population appears to be quite heterogeneous with a large range in response magnitude compared to adults (Supplementary Fig. 2b, c). Despite having a different profile than both the adult COVID-19 and the negative populations, the infected pediatric population cannot be clearly discriminated from these two groups using antibodies to structural proteins.

We then selected accessory protein antibodies as combinations to investigate the relevance of unique markers. Previously we showed that, ORF3d, ORF8, and N antibodies, can discriminate accurately COVID-19 adults from negative controls^24^. The (N, ORF3d, ORF8) cluster of points can accurately allow the positive discrimination of the pediatric COVID-19 cases from the negatives (Fig. 3c). In the (N, ORF8; x, y) plane, the negative population is separated from the pediatric positive one by two-segments of straight lines (equations of 830*log (N) + 0.3843*ORF8 = 4801 and −350*log (N) + 1.036*ORF8 = 790), with all pediatric positive samples (red dots) represented above or on these lines, and only one negative sample (gray triangle) being above these lines (specificity of 96.9% and sensitivity of 100% for pediatric cases). Of note, this plane did not allow an accurate discrimination of the adult positive population (blue dots) with the negative one (gray triangles), as some adult samples (*n* = 8 of 71) were found below these lines, with the negatives. Interestingly, these 8 samples were early time-point samples (mean time-point sampling: 2.6 days of infection).

Furthermore, the plane (ORF8, ORF3d; y, z) and a two-segment delineation (equations of 0.035*ORF3d + 0.1334*ORF8 = 409.284 and 0.074*ORF3d + 0.0437*ORF8 = 221.812) separated the negative samples from all the adult and pediatric positive ones (100% sensitivity and 100% specificity), therefore the combined use of ORF3d and ORF8 most accurately discriminates infected samples from uninfected controls. While the combination of ORF3d and N did not allow any clear discrimination of any of the 3 populations: pediatrics infected, adults infected, and negatives.

Therefore, using the (N, ORF3d, ORF8) cluster analysis, the pediatric COVID-19 population resembles a COVID-19 adult population when these markers are taken together only, and can be discriminated from negative pre-pandemic controls. Importantly, this is the only combination that allowed us this discrimination of infected samples, as other parameter combinations (e.g., (S1, S2, S2′) in Fig. 3, (N, E, M) in Supplementary Fig. 2) and combinations of antibodies to accessory proteins) were also tested and represented as clusters of points but did not discriminate pediatric samples. Our combined antigen analysis (Fig. 2i) and these various data cluster analyses show that the antibody specificity of the COVID-19 children population is distinct from infected adults.

To test the hypothesis that the antibody specificity to structural and accessory viral proteins drives the distinct profile of the pediatric population, we undertook a principal-component analysis (PCA) of antibodies to the 14 SARS-CoV-2 antigens for the full dataset (from Figs. 1 and 2). Dimension (principal component) 1 and 2 explained, respectively, 20.6% and 17.7% of the total variances from all the 14 antibody types (Supplementary Fig. 3 and Fig. 3d, e). Antibodies to accessory proteins ORF3d, NSP1, ORF3a, ORF8, ORF7a had high correlation values (Supplementary Table 2), reflecting that antibodies to structural proteins do not solely drive the principal component 1. Particularly, contributions of ORF3d, NSP1, ORF3a, ORF8 were the highest in Dimension 1 (Dim1, Fig. 3d, e and Supplementary Table 3). Moreover, PCA showed that ORF3d and ORF7a antibodies highly contributed to the differences seen in both dimensions (Fig. 3e, f) highlighting their discrimination in the serological response.

Strikingly, the PCA revealed that pediatric COVID-19 antibody response was also intermediate between COVID-19 adults and negatives (Fig. 3g). Indeed, the normal-probability representation of the 3 populations showed that only 31.5% of the pediatric patients overlapped with the ellipse of the COVID-19 adults and only 4.72% overlapped with the ellipse of negative controls (Fig. 3g). Figure 3h and statistical comparison of the 2-dimensional distributions of the pediatric and adult groups “asymptomatics”, “mild”, and “severe” revealed that the distribution of the population of severe adult patients was significantly different than that of the adult mild or asymptomatic populations (*p* = 0.027 for severe versus asymptomatic adult cases, and *p* = 0.011 for severe versus mild adult cases with the Kolmogorov–Smirnov test^36,37^. Overall, the PCA showed that adults with severe symptoms had a distinct PC value distribution than adults with mild symptoms (Fig. 3h), which may be driven by these differences in S1, S2′, and ORF8.

Further analysis on sex, infection time-point, and neutralization data (PRNT90) values showed they were not significant factors in discriminating the antibody specificity data (Supplementary Fig. 3). Therefore, the differences in the observed SARS-CoV-2 antibody responses are primarily explained by the age of patients (pediatric COVID-19, adult COVID-19 or pre-pandemic negative controls), and clinical symptoms.

### No difference in antibody responses between symptomatic and asymptomatic COVID-19

**Fig. 4:**
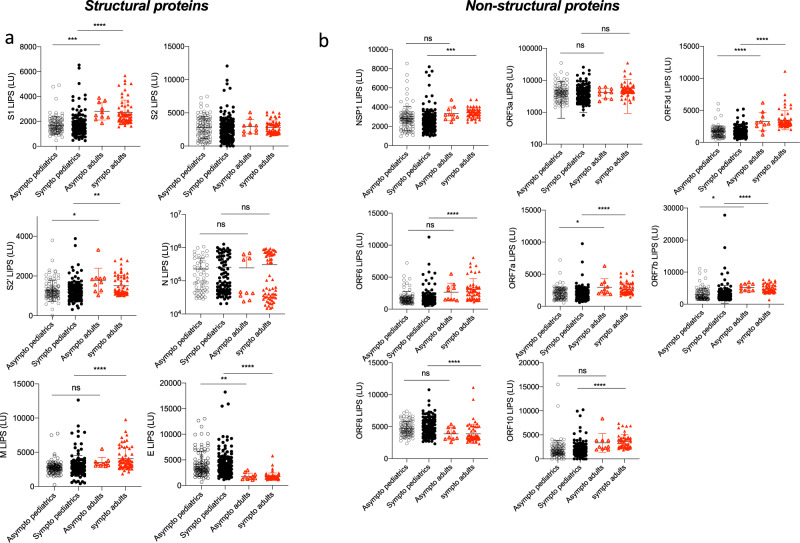
Asymptomatic and mildly symptomatic children do not display different antibody responses. Pediatric and adult samples were stratified according to the symptom score of the patients (asymptomatic “asympto” (pediatric COVID-19 *n* = 98, adults COVID-19 *n* = 10) versus symptomatic “symptom” (pediatric COVID-19 *n* = 156, adults COVID *n* = 52)), data from Figs. 1 and 2 were analyzed according to “asympto” and “sympto”. **a** Antibodies against the SARS-CoV-2 structural proteins S1, S2, S2′, N, E, and M by LIPS. Significant *p* values for asympto pediatrics versus asympto adults are S1 *p* = 0.0005, S2′ *p* = 0.0221, E *p* = 0.0071. Significant *p* values for sympto pediatrics versus sympto adults are S1 *p* < 0.0001, S2′ *p* = 0.0016, M *p* < 0.0001, E *p* < 0.0001. **b** Antibodies against SARS-CoV-2 NSP1 (in ORF1ab), and all other ORFs (ORF3a, ORF3d, ORF6, ORF7a, ORF7b, ORF8, and ORF10). Significant *p* values for asympto pediatrics versus asympto adults are ORF3d *p* < 0.0001, ORF7a *p* = 0.0358, ORF7b *p* = 0.0208. Significant *p* values for sympto pediatrics versus sympto adults are NSP1 *p* = 0.0001, ORF3d *p* < 0.0001, ORF6 *p* < 0.0001, ORF7a *p* < 0.0001, ORF7b *p* < 0.0001, ORF8 *p* < 0.0001, ORF10 *p* < 0.0001. Two-sided *P* values were calculated using the Mann–Whitney U test. * shows statistical significance between COVID-19 patients versus negative controls. *p < 0.05, **p < 0.01, ***p < 0.005, ****p < 0,0001. All data represents individual time point LIPS responses and mean ± stdev.

To assess the potential effect of antibodies to structural and non-structural proteins of SARS-CoV-2, we further stratified data (from Figs. 1 and 2) into symptomatic (including mild (WHO score 1–3) and severe (WHO score 4) and asymptomatic (WHO score 0) for both the adult and pediatric cohorts (Fig. 4). We found no differences in antibody responses between asymptomatic versus mild COVID-19 children for all 14 antigens. The same trend was observed in adults (Fig. 4a, b). More importantly M, NSP1, ORF6, ORF8, and ORF10 antibody levels in asymptomatic children versus asymptomatic adults were not significantly different (*p* = 0.3676, *p* = 0.5216, *p* = 0.1276, *p* = 0.2775 and *p* = 0.0521, respectively, Fig. 4), while symptomatic adults had an upregulated antibody response to these antigens compared to symptomatic children (*p* < 0.0001 for all antigens, except *p* = 0.0001 for NSP1, Fig. 4).

### Antibody specificity at early infection and long-term stability

**Fig. 5:**
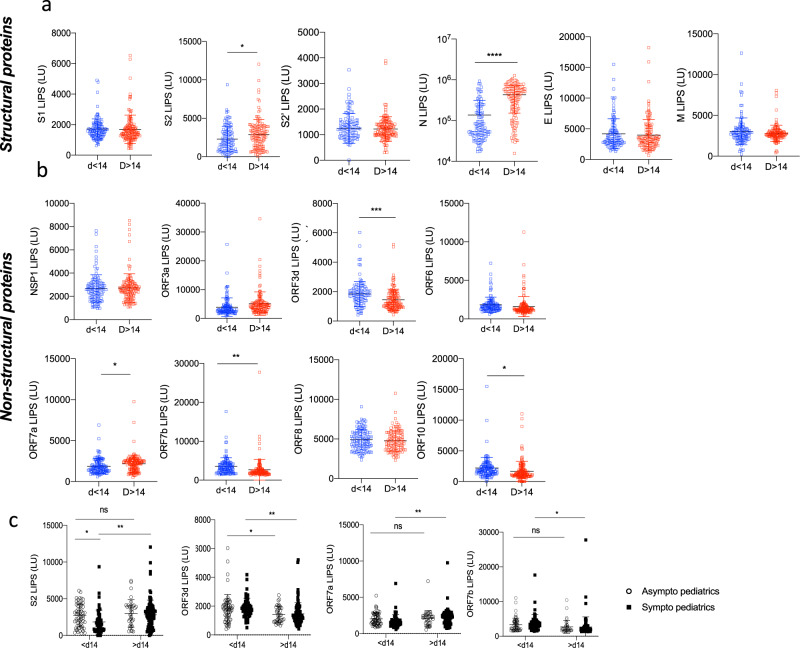
Unique antibody specificities are characteristic of early time-point samples (< day 14). Pediatric samples were stratified according to the time-point of collection, and data from Figs. 1 and 2 were analyzed according to acute (<day 14, *n* = 119) and later time-points (≥day 14, *n* = 135). **a** Antibodies against the SARS-CoV-2 structural proteins S1, S2, S2′, N, E, and M by LIPS. Significant *p* values for <day 14 versus ≥day 14 are S2 *p* = 0.0287, N *p* < 0.0001. **b** Antibodies against NSP1 (in ORF1ab), and all other ORFs (ORF3a, ORF3d, ORF6, ORF7a, ORF7b, ORF8, and ORF10). Significant *p* values for <day 14 versus ≥day 14 are ORF3d *p* = 0.0001, ORF7a *p* = 0.048, ORF7b *p* = 0.0093, ORF10 *p* = 0.0288. Two-sided *P* values were calculated using the student *t* test. * shows statistical significance between acute time-point pediatric COVID-19 patients versus late time-point pediatric COVID-19 patients. **c** Stratification of S2, ORF3d, ORF7a, and ORF7b antibody levels measured by LIPS according to symptoms (“asympto” for asymptomatic, “sympto” for symptomatic) and time-point of sampling (prior day 14 or after day 14). Significant *p* values for <day 14 asympto versus <d14 sympto are S2 *p* = 0.0172. Significant *p* values for asympto <d14 versus asympto >d14 are ORF3d *p* = 0.0309. Significant *p* values for sympto <d14 versus sympto >d14 are S2 *p* = 0.0021, ORF3d *p* = 0.0044, ORF7a *p* = 0.0066, and ORF7b *p* = 0.0123. Two-sided *P* values were calculated using the Mann–Whitney U test. * shows statistical significance between symptomatic and asymptomatic samples or between early and late samples *p < 0.05, **p < 0.01, ***p < 0.005, ****p < 0.0001. All data represents individual LIPS responses and mean ± stdev.

We previously observed that the SARS-CoV-2 antibody responses can vary in magnitude and specificity in adults between acute and convalescent to memory time-points^24^. To study the effect of time on the pediatric SARS-CoV-2 antibody specificity, we stratified pediatric responses of all 254 samples (Figs. 1 and 2) by early (<d14) versus later (≥d14) time-points of infection (Fig. 5). S2, N, and ORF7a specific antibodies were significantly increased after day 14 post symptom onset. In contrast, ORF3d and ORF7b antibodies elicited a higher antibody response prior to day 14 (Fig. 5b). Finally, responses to structural proteins S1, S2′, M, and E and accessory proteins NSP1, ORF3a, ORF6, and ORF8 were comparable before and after day 14 (Fig. 5a, b). Further stratification of samples according to time-point of sampling and symptoms was then performed (Fig. 5c) and showed significant differences for symptomatic pediatric patients for convalescent samples for key antigens, S2, ORF3d, ORF7a, and ORF7b.

**Fig. 6:**
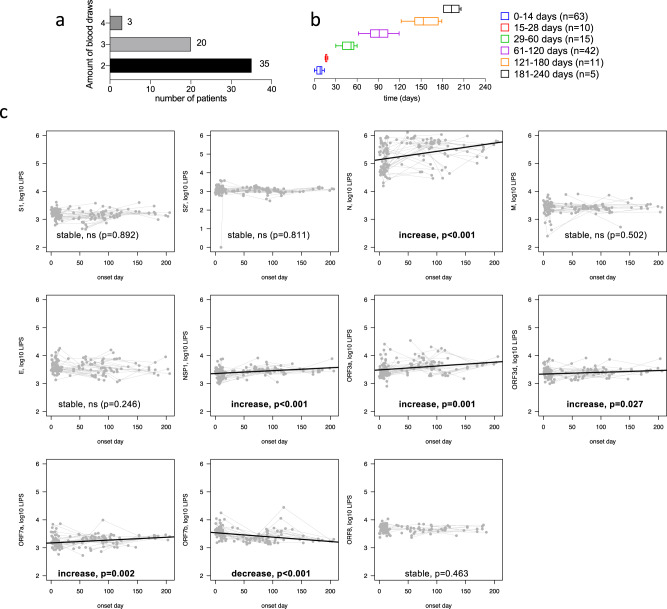
Longitudinal stability of antibody responses for structural and non-structural SARS-CoV-2 proteins in COVID-19 children. **a** Number of longitudinal patients with either 2, 3, or 4 blood draws from 58 pediatric COVID-19 cases. **b** Sample collection time-line (days post infection). For each plot, whiskers are minima to maxima, and center is mean, no percentile is shown. Detailed minima, maxima, mean ± S.D. are as follows: Days 0–14:0, 14, 7.4 ± 4.02, days 15–28: 15, 19, 17.0 ± 1.51, days 29–60: 30, 60, 48.7 ± 9.78, days 61–120: 62, 119, 91.7 ± 15.42, days 121–180: 122, 179, 156.3 ± 19.96, days 181–240: 181, 206, 192.7 ± 11.33. **c** A linear trend on log_10_ LIPS values was fitted for longitudinal samples for S1, S2′, N, M E, NSP1, ORF3a, ORF3d, ORF7a, ORF7b, ORF8 (*n* = 58 pediatric COVID-19 patients). Linear mixed-effects models were fitted to test the trend of the antibody responses. The trend is specific to each SARS-CoV-2 protein and hence adjustment for multiple comparisons is not needed.

To further confirm the stability of SARS-CoV-2 specific antibodies, we used 146 longitudinal paired samples of 58 pediatric patients that had either 2, 3 or 4 blood draws (Fig. 6a). The time frame of sampling ranged from 0 to 206 days post-symptom onset, with most samples from <14 days (*n* = 63), or long-term memory samples after day 60 (*n* = 58) (Fig. 6B). Using a linear mixed-effects model, we determined that antibody responses to structural proteins S1, S2, S2′, M, and E were stable over time, whereas N was significantly increased (*p* < 0.001) (Fig. 6c). Furthermore, antibodies towards non-structural proteins NSP1, ORF3a, ORF3d, and ORF7a also significantly increased over time (*p* < 0.001, *p* = 0.001, *p* = 0.027 and *p* = 0.002, respectively), while ORF6, ORF8, and ORF10 were stable (Fig. 6c). Only ORF7b antibody response significantly decayed longitudinally at a slow rate (equation of: log10 LIPS = 3.5539−0.0016 * day after onset, *p* < 0.001, Fig. 6c). To determine whether the slope of each serological marker was related to symptoms we compared asymptomatic and symptomatic patients, but no significant differences were found (*p* > 0.05 for all) (Supplementary Fig. 4).

### Effect of age within the pediatric population

Further stratification of the pediatric cohort according to the age of the patients was performed but did not reveal a specific pattern for any age-groups (0–2, >2–10, >11 years old) as shown by the representation of the population as a cluster of points for the most relevant (N, ORF8, ORF3d) antibody combination (Supplementary Fig. 5).

### Circulating cytokine levels

**Fig. 7:**
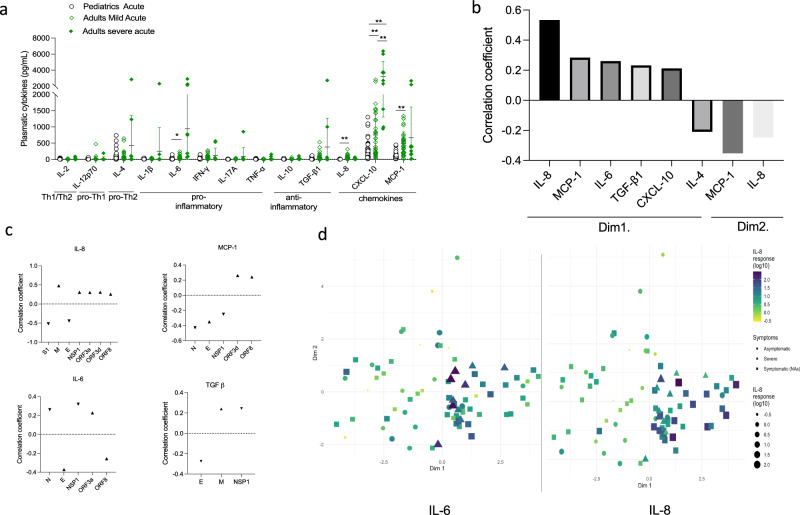
IL-8 and MCP-1 contribute the biggest determinate of the COVID-19 antibody responses, and IL-6 and IL-8 are associated with disease severity of antibody responses. **a** Plasma levels of 13 circulating cytokines in pg/mL for acute samples (<day 7) for pediatric cases (*n* = 35) or adults mild (*n* = 34) and severe cases (*n* = 10): IL-2, IL-12p70, IL-4, IL-1β, IL-6, IFN-γ, IL-17A, TNF-α, IL-10, TGF-β1, IL-8, CXCL-10, MCP-1. Data represent individual cytokine responses and mean ± stdev. For pediatrics versus adults mild: *p* = 0.0019 for IL-8, *p* = 0.0078 for CXCL10, *p* < 0.0001 for MCP-1. **b** Highest correlation coefficient of cytokines with Dim-1 and Dim-2 of the antibody responses from Fig. 3. **c** Correlation coefficients of specific antigens with the cytokines of interest IL-8, MCP-1, IL-6, and TGF-β. **d** Association of IL-6 and IL-8 levels with clinical symptoms in Dim-1 and Dim-2 of the antibody responses from Fig. 3. Severe (triangles) and mild (square) cases are darker than asymptomatic cases and located on the right of the graph meaning that they strongly associated with Dim-1 ((*p* < 0.05 and *p* < 0.0001, respectively). Data were analyzed using the two-sample Wilcoxon test for comparison of the responses between asymptomatic and symptomatic cases.

Severe COVID-19 has been linked with a cytokine storm^38^ and some cytokines are now the targets of COVID-19 therapies, i.e., IL-6 for which a monoclonal antibody therapy is being investigated for the treatment of critically ill COVID-19 patients^39^. Because children mainly suffer from mild COVID we sought to investigate the relationship between antibody production and cytokine profile in this population. We selected pro-Th1/Th2, pro/anti-inflammatory cytokines, and chemokines that had also been described as early prognostic markers of severe COVID-19^40,41^ and therefore play an important role in shaping the adaptive immune priming. Cytokines of interest were quantified at acute stages of infection (<day 7 from 36 mild COVID-19 children and adults and 10 severe adults from the total cohort were measured in the plasma samples with a Cytokine Bead Array to determine the relationship between inflammation and seroconversion. Levels of plasma chemokines IL-8, CXCL10, and MCP-1 were significantly elevated in mild adults compared to pediatric cases (*p* = 0.0019, *p* = 0.078 and *p* < 0.0001, Fig. 7a). Of note, CXCL-10 levels were also found significantly elevated in severe adults versus mild adults and versus pediatric cases (*p* = 0.0067 and *p* = 0.0025). The pediatric population also showed reduced levels of the pro-inflammatory cytokine IL-6 with a 3-times reduction versus adult mild cases (mean ± stdev: 13.86 ± 27.7 versus 41.21 ± 57.1, *p* = 0.0394).

To evaluate the importance of each of the cytokines and antibody specificity, we performed a PCA of the cytokine levels and antibody responses and found that IL-8 and MCP-1 were the biggest contributors to the overall antibody responses in Dim.1 and Dim.2 (*p* < 0.05) (Fig. 7b). Moreover, IL-6, TGF-β1, CXCL-10, and IL-4 all correlate with Dim.1, with IL-4 being the only cytokine correlating negatively (Fig. 7b). Our focus was then brought to the correlation of each specific antigen with the cytokines of interest IL-8, MCP-1, IL-6, and TGF-β1 (Fig. 7c). Amongst structural proteins, responses to S1 and E correlated negatively with IL-8 responses, while M correlated positively with these cytokines (Fig. 7c, *p* < 0.0001, *p* < 0.001 and *p* < 0.001, respectively). Furthermore, antibodies to accessory proteins NSP1, ORF3a, ORF3d, and ORF8 positively correlated with IL-8 responses (Fig. 7c, *p* < 0.05, *p* < 0.05, *p* < 0.05 and *p* < 0.0001, respectively). ORF3d and ORF8 responses also correlated positively with MCP-1 levels (Fig. 7c, *p* < 0.05). On the other hand, antibodies to the structural protein E correlated negatively with all four cytokines of interest (*p* < 0.0001 for IL-8, MCP-1, and IL-6, and *p* < 0.05 for TGF-β1). Importantly, E antibodies were elevated in infected children (Fig. 1f).

Furthermore, a positive correlation was found between N, NSP1, and ORF3a antibody responses and IL-6 (Fig. 7c, *p* < 0.05). Finally, we analyzed if a pattern related to these cytokines could predict disease outcome, and found that IL-6 and IL-8 levels were strongly associated in Dim1 with disease severity (*p* < 0.05 and *p* < 0.0001, respectively, Fig. 7d). This highlights the close correlation of antibody responses toward the driving antibodies of Dim.1 (ORF3d, NSP1, ORF8, and ORF3a) and cytokine responses (particularly for IL-6 and IL-8) with disease outcome.

## Discussion

Young children account for only a small percentage of reported and medically attended COVID-19 infections^9^. This difference is likely contributed to by differences in host responses between children and adults that ultimately drive adaptive immunity. We present herein a comprehensive study of the magnitude, specificity, and duration of SARS-CoV-2 specific antibodies in children.

Our data show that children produce antibodies to some accessory proteins at reduced levels compared to adults (namely S1, M, ORF3a, ORF7b), and that only one accessory target induced an increased antibody response: ORF8. Overall, we found a significant diverse distribution of the antigenic targets in children and adults. These diverse levels of antibodies to structural and accessory ORF proteins may reflect different virus pathogenesis in children compared to adults. Viral loads have been shown to be comparable in children and adults, which may reflect similar levels of viral replication^2^. The higher levels of ORF8 antibodies in the pediatric population could be of particular interest as this glycoprotein has been shown to downregulate MHC-I molecules^30^ and seems to play an important role in viral pathogenesis^31^. Similarly, we also observed that severe adult cases had lower levels of ORF8 antibodies than mild adult cases, and that infected children had elevated ORF8 antibodies, therefore ORF8 antibodies may function as a positive immune correlate. Whether ORF8 antibodies found in children can block some of the deleterious functions of this protein remains to be determined by functional studies. Higher ORF8 antibodies in children is also in line with reduced cellular immunity^42^, and may also reflect higher expression of ORF8 during pediatric infection that is known to reduce MHC-I presentation^30^.

Antibodies to the structural protein E were also present in higher proportions in children than adults, and the E protein is notorious for high turnover due to its pivotal role in viral propagation (reviewed in ref. ^43^). Therefore the extent and speed of virus replication in pediatric cells may be different to adults due to differences in innate pathway activation^44^ leading to antibody priming being elevated in children for E, which warrants further investigation.

For the antibodies to Spike sub-units, the (S1, S2′, S2) cluster reveals that the children population resembles a negative pre-pandemic population and not a COVID-19 adult one. A recent study describes a lower anti-S IgG, IgM, IgA in the pediatric population which correlates with our findings^45^. One explanation for the clinical difference between children and adults is that the pre-existing immunity against seasonal human CCoV that cross-reacts with SARS-CoV-2 is higher in children, as they have a higher infection rate of seasonal CCoV than adults^46^. Individuals exposed and unexposed to SARS-CoV-2 have cross-reactive antibodies against the proteins of SARS-CoV-2 and seasonal CCoVs^25,47^. Moreover, because circulating CCoVs have a higher homology to SARS-CoV-2 structural proteins than non-structural proteins (if they exist)^48,49^, we expect a higher cross-reactivity for structural proteins based on pre-existing immunity. SARS-CoV-2 infection back-boosts antibodies against conserved epitopes, including the relatively conserved fusion peptide of the Spike S2 subunit^25,47^. In our hands, COVID-19 children and adults had comparable levels of S2 antibodies, contrary to S1 and S2′, which shows a possible effect of pre-existing CCoVs immunity for more conserved domains of S such as S2. Shrock et al. used VirScan, a DNA bacteriophage microarray, to investigate cross-reactivity between SARS-CoV-2 and CCoV in COVID-19 patients^25^. They identified cross-reactive epitopes and found that cross-reactivity was weaker in severe patients than in mild patients, but samples from children were not included in this study.

Our observations of lower Spike antibodies in COVID-19 children may indicate that there may be lower sensitivity of serological detection for SARS-CoV-2 when using assays based on S alone^50^, leading to an underestimation of SARS-CoV-2 exposed children. S antibodies have been reported in lower magnitude in the majority of mild adult infections, with higher levels being produced in severe cases^51^, which is consistent with our data on low S antibody levels in children which were also asymptomatic or mild clinical scores. Low antibody levels and low affinity have been associated with Antibody-Dependent Enhancement by facilitation of viral uptake by host cells^52^, it has now been demonstrated that binding of antibodies to the N-terminal domain (NTD) of S enhances infectivity^53^, meaning that lower S-NTD antibody prevalence in children could be advantageous.

The combinatory use of ORF8 and ORF3d antibodies has been shown to be a highly specific and sensitive tool for COVID-19 serology diagnostic^24^, and appears here as an accurate tool also for the pediatric population. In line with our results, ORF8 has recently been reported as an immunodominant antigenic site with high sensitivity for the serodiagnosis of mild and severe COVID-19 children^54^. The plane (ORF3d/ORF8) in the cluster of points (N, ORF3d, ORF8) reveals that children samples have specific combinatory values of these two antibodies that is consistent with adult populations, and that makes them distinguishable from uninfected controls. The PCA of our dataset confirmed further the importance of antibodies to accessory proteins in characterizing the pediatric samples, notably ORF3d, NSP1, ORF3a, ORF8. Whether these antibodies to accessory proteins play a role in the virus infectivity or in the pathogenesis of the disease and in the milder outcome of SARS-CoV-2 infection in children presents further questions for investigations.

We report in children diverse antibody profiles in early versus late samples and the maintenance or increase of all antibodies to structural and accessory proteins, except ORF7b antibodies, for at least 6 months post-infection. Many factors play a role in antibody long-term persistence, such as antigen release, antigen presentation, induction of a germinal center reaction, and a memory B cell pool^55^. Additional studies on viral proteins release, their roles, and their specific B cells are needed to fully understand the pattern of antibody specificity in children.

To complement serological data and to understand the link between the inflammatory response and the distinct pattern of antibody specificity observed in children, circulating cytokines were measured in samples from acute time-points (<day 8). In our hands, IL-8 and MCP-1 correlated with the overall antibody specificity observed in our cohort, and particularly with antibodies to accessory proteins, including ORF3d and ORF8, reflecting the potential role of these targets in the shaping the adaptive host-pathogen response. These chemokines have been identified by others as determinant in the cytokine storm observed in COVID-19 patients^40^, and have been shown to predict disease severity and outcome^41^. High serum levels of IL-6, IL-8, and MCP1^41,56^ have been correlated with poor prognosis, hence the identification of potential antibody associations and levels could also inform on disease outcome and mechanisms of viral pathogenesis. Pediatric patients have been shown to have lower disease burden and subsequently have lower cytokine levels compared to adults for IL-8, IL-6, and MCP-1, consistent with previous findings^41,56^. Moreover, blockade of ORF8 signaling has been shown to reduce production of hyper-inflammatory cytokine^57^. Interestingly ORF8 antibodies correlated negatively with IL-6, which independently predict disease severity^41^. Whether these antibodies can play a beneficial role in reducing the cytokine storm and the overall viral infection needs to be determined, as ORF8 has been shown to play a role in the pathogenesis of the virus^30,31^.

Structural protein E correlated negatively with all cytokines tested and has been shown to not elicit an antibody response in COVID-19 mild adults^24^ but does contribute to 10.99% of the magnitude of the pediatric antibody response pointing to the different expression of E during pediatric infection. Indeed, E is a TLR2 ligand for SARS-CoV-2 that can induce pro-inflammatory cytokines often associated with severe COVID-19^58^, hence the potential of anti-E antibodies blocking that interaction in children should be further investigated and potentially considered as a therapeutic tool in severe COVID-19.

Our cohort did not include any case of the very rare Multi-Inflammatory System in Children (MIS-C). In our hands, symptomatic (mild) children have significant differences in antibody levels versus symptomatic adults for antibodies to all accessory proteins tested (excluding  ORF3a) which suggests that these accessory markers could play a role in infection control or infectivity. One study reported that no distinct antibody response was observed between MIS-C and mild or asymptomatic children^45^, though the authors only measured S and N antibodies. More recently, Ravichandran et al. described the SARS-CoV-2 immune repertoire in MIS-C versus mild and severe pediatric COVID-19 cases and identified a diverse antibody-signature associated with disease severity in children^54^, highlighting the need for further studying the antibody specificity in this population to determine mechanisms for pathogenesis.

It is possible that the interest in antibodies to SARS-CoV-2 internal proteins will grow with the rollout of sub-unit Spike only and whole inactivated virion vaccines, in order to allow the distinction between SARS-CoV-2 past infection and vaccination in specific populations and to create an estimated date of exposure given the unique rate of waning of different specificities. Inactivated vaccines represent the greatest proportion of COVID-19 vaccine doses^59^, and N remains an immunodominant antibody response in CoronaVac whole inactivated virus-vaccinated individuals^60^. So far, S and N-based serology have been inadequate for this^18,61^, and in our hands ORF8 could distinguish between vaccinated (both Spike mRNA and whole inactivated virus vaccines) and infected convalescent controls (Supplementary Fig. 1f). The emergence of certain viral mutants such as the ORF8 truncations^31^ or recent ORF3d deletions^62^ could modify the contributions of certain ORFs to virus pathogenesis and their utility as serological markers of infection in the future. These unique antibody responses could also be useful for epidemiology studies on the insurgence of different strains of the virus due to higher conservation rates than surface proteins. However, combined multivalent serology may be needed in the case of deletion variants such as Alpha B1.1.7 for ORF8 and Beta B1.35.1 for ORF3d (reviewed in ref. ^63^). Furthermore, the detection of various isotypes of antibodies for internal proteins such as IgM and IgA could also be relevant to assess the class switching, kinetic and Fc-related functions of the responses to non-surface proteins. Previously, a study using a SARS-CoV-2 proteome microarray enabled the detection of IgG and IgM to a wide-range of viral proteins, and identified alternative proteins NSP5 and ORF9b as significant antibody targets^26^.

In conclusion, we report the description of a more diversified antibody specificity in the COVID-19 children population compared to adults, with a sustained humoral response to all accessory proteins of the virus. This study of antibody spectrum provides insights into the importance of the breadth of antibody responses which differs between children and adults possibly due to differences in virus replication, expression levels, and different immune responses in children towards key viral proteins, ORF8 and E. Our study calls for improved SARS-CoV-2 diagnostics for the pediatric population to move beyond the spike and utilize additional antigenic targets that trigger larger responses that are more stable with time. This will become more important in the near future with the up-coming roll-out of vaccines in the pediatric population where natural infection and vaccination will need to be distinguished.

## Methods

### Patients and samples collection

Our study enrolled a total of 122 children patients and 71 adult unvaccinated patients based on recruitment of available patients with RT-PCR confirmed COVID-19 infection in Hong Kong and in the USA. We used a total of 254 COVID-19 children plasma samples including 146 longitudinal samples from 58 subjects with 2 to 4 sampling time points, and 119 early time-points samples (< day 14). Samples were used from children (mean ± stdev: 39 ± 47 days, range: 0–206 days) and adults (mean ± stdev: 20 ± 23 days, range: 0–123 days), with the sample day defined as day post-symptom onset or RT-PCR confirmation for asymptomatic cases through contact tracing or quarantine. The COVID-19 patient study was approved by the institutional review board of the respective hospitals, viz. Kowloon West Cluster (KW/EX-20-039 (144-27)), Kowloon Central/Kowloon East cluster (KC/KE-20-0154/ER2) and HKU/HA Hong Kong West Cluster (UW 20-273, UW20-169), Joint Chinese University of Hong Kong-New Territories East Cluster Clinical Research Ethics Committee (CREC 2020.229), and the Human Research Protection Office at Washington University in St. Louis, USA (IRB reference number 202007097). All of the patients provided informed consent.

The negative control plasma samples used in this study were from Hong Kong blood donors collected from June to August 2017 (prior to the emergence of COVID-19), used a total of 48 plasma samples including negative pediatric samples (*n* = 20) and negative adult samples (*n* = 28). The collection of negative control blood donors was approved by the Institutional Review Board of The Hong Kong University and the Hong Kong Island West Cluster of Hospitals (approval number: UW16-254). All of the donors provided informed consent. Plasma samples were collected from heparinized blood. All samples from COVID-19 patients or negative controls were heat-inactivated prior to experimental use at 56 °C for 30 min. Heat-inactivated and non-heat-inactivated samples were initially tested in the laboratory and by others^64,65^. Details on the sample cohort are presented in Table 1.

### Luciferase immunoprecipitation system (LIPS) assay

The LIPS assay is a liquid phase immune-assay allowing the unbiased quantification of antibodies by measuring luminescence emitted by the reporter enzyme Renilla luciferase (Ruc) fused to an antigen of interest, expressed by the pRen2 vector in mammalian cells. Luciferase, a light-producing enzyme is used as a reporter. The construction of *Renilla* luciferase (Ruc) chimeric genes involves the mammalian expression vectors pREN2 in which the antigen of interest is fused in-frame with Ruc. Protein A/G beads capture the immunoglobulin-antigen complexes. Protein A/G binds predominantly to the four isotypes of IgG but can also bind, to a much lesser extent, to IgA and IgM. Therefore, our data primarily represents plasma IgG. The use of LIPS in the diagnostic of SARS-CoV-2 infection has been published by us and others (for the 14 antigens tested in the present study^24^, for Spike and Nucleocapsid^66–68^).

Based on previous studies describing the structure of the SARS-CoV-2 genome^49,69^, an extensive panel of 14 proteins (S1, S2, S2′, E, M, N, NSP1, ORF3a, ORF3, ORF6, ORF7a, ORF7b, ORF8, ORF10) was chosen for antibody testing by LIPS. Primers for the amplification of SARS-CoV-2 proteins were designed (see protein ID and primers sequences in Supplementary Table 1). Primers and cloning for the amplification of SARS-CoV-2 proteins were as previously described^24^. Constructs with pREN2-Renilla luciferase plasmid containing the SARS-CoV-2 antigen of interest were transfected into Cos1 cells using Fugene 6 (Promega) as per manufacturer’s instructions. Cells were harvested 48 h later, lysed and sonicated, and (Ruc)-antigen yields were measured using a Luminometer plate reader (PerkinElmer) according to the protocol of Burbelo et al.^64^ as previously described, with the following modifications^24^.

Briefly, (Ruc)-antigen (at an equal concentration for each antigen at 10^7^ per well) and plasma (heat-inactivated and diluted 1:100) were incubated for 2 h with shaking at 800 rpm. Ultralink protein A/G beads (Thermo-Fisher) were added to the (Ruc)-antigen and serum mixture in a 96-deep-well polypropylene microtiter plate and incubated for 2 h with shaking at 800 rpm. The entire volume was then transferred into HTS plates (Millipore) and washed as previously described. The plate was read using QUANTI-Luc Gold substrate (Invivogen) as per manufacturer’s instructions on a Wallac MicroBeta JET luminometer 1450 LSC & Luminescence counter and GLOMAX v1.7.1 software for analysis (Promega). Experimental controls include no plasma blank wells with (Ruc)-antigens and negative control serum from healthy donor plasmas collected prior to the COVID-19 pandemic (Table 1). The background corresponds to the LU signal from each Ruc-fusion antigen with protein A/G and substrate with no plasma. Responses were considered negative when they were not significantly elevated compared to negative pre-pandemic controls.

### Enzyme-linked immunosorbent assay

Total IgG were measured in plasma samples using the Total humanprot IgG ELISA kit (Thermo-Fisher) at a final dilution of 1:500,000 according to manufacturer’s instructions.

### Cytokine bead array

For the measurement of cytokines in the plasma, the Human essential immune 13 cytokines LegendPlex (Biolegend) panel was used and included: IL-4, IL-2, IP-10, IL-1β, TNF-α, MCP-1, IL-17, IL-6, IL-10, IFN-γ, IL-12p70, IL-8, TGF-β1. Standard wells were run in duplicates. Samples were acquired by flow cytometry on a FACS Attune (Invitrogen) and analyzed with LegendPlex software as per manufacturer’s instructions.

### Analysis and representation of the dataset as clusters of points

The SARS-CoV-2 antibodies dataset has been analyzed through the free software ConTeXt, with LuaMetaTeXengine (version 2020.05.18) developed by Hans Hagen (http://www.pragma-ade.nl) which uses TeX, Metapost, and Lua to obtain the 3D clusters of points shown in Figs. 3a–c, 7c and Supplementary Fig. 2. In the clusters of points, each sample is represented according to 3 parameters in the 3 axes (x, y, z). This analysis of the dataset considers a combination of three different parameters taken together. For clarity, only the first 144 COVID-19 pediatric samples of the dataset are represented in the clusters of points, along with the total COVID-19 adult samples (*n* = 71) and negatives (*n* = 48).

In the cluster (N, ORF3d, ORF8), the equations of the red lines are (1) in the plane (N, ORF8): 830*log (N) + 0.3843*ORF8 = 4801 and −350*log (N) + 1.036*ORF8 = 790, and (2) in the plane (ORF3d, ORF8): 0.035*ORF3d + 0.1334*ORF8 = 409.284 and 0.074*ORF3d + 0.0437*ORF8 = 221.812. These straight lines allow the most accurate discrimination between negative controls and positive adult populations.

### Principal component analysis

The LU for 14 antigens were log-scale transformed (the negative and zero values in the dataset were replaced by 1) prior to PCA analysis. The missing values in the dataset were estimated by a probabilistic model^70^. The probabilistic model is tolerant to amounts of missing values between 10% and 15% which is fit for our data. The missing data were estimated using pcaMethods (version 1.80.0)^71^. The completed data were standardized (scaled) before input in standard PCA (using *FactoMineR* (version 2.4)^72^. The PCA results were extracted and visualized using factoextra (version 1.0.7)^73^.

### Statistics and reproducibility

GraphPad Prism version 10 software (San Diego, CA) was used for statistical analysis. All experiments were repeated twice independently. Antibody levels are presented as the individual responses and geometric mean ± standard deviation (stdev). Ordinary one-way ANOVA with Tukey’s multiple comparison test was performed to compare the pediatric, adult, and negative populations in Figs. 1 and 2, and the early and late samples in Fig. 5. For Fig. 2j, percentages were calculated by dividing each mean antibody value by the sum of the total antibody responses (including or excluding N responses), and compared using a Chi-square test between the “observed” (pediatric) versus “expected” (adult) distributions.

For Fig. 6c and Supplementary Fig. 4, a linear mixed-effects model was fitted to account for correlated responses for the longitudinal samples dataset. Log_10_ LIPS was used for the analysis (as dependent variable) to reduce the impact of extreme values/non-normality. For Supplementary Fig. 1, the distributions showed in the pie-charts were compared using a Chi-square test between the “observed” (pediatric) versus “expected” (adult) distributions.

### Reporting summary

Further information on research design is available in the Nature Research Reporting Summary linked to this article.

## Supplementary information

Supplementary Information

Peer Review File

Reporting Summary

## Data Availability

The raw data that support the findings of this study included as supplementary files, from which PCA analysis is derived from (Fig. 3d–h, and 7b-d). Data from LIPS and ELISA IgG responses with background subtracted are indicated in all figures are shown.
