# Supplementary material for: SARS-CoV-2 accessory proteins reveal distinct serological signatures in children
Source: Nat Commun. 2022 May 26;13:2951. doi: 10.1038/s41467-022-30699-5 (PMC9135746; doi:10.1038/s41467-022-30699-5)
Supplement: Supplementary file 1 — Supplementary Information [file 41467_2022_30699_MOESM1_ESM.pdf]

Supplemental methods

SARS-CoV-2 specific ELISA

Plates (Nunc MaxiSorp, Thermofisher Scientific) were coated with 250 ng/ml SARS-CoV-2 S or N protein (SinoBiological) overnight or 300 ng/mL ORF8[1] (Masashi Mori, Ishiwaka University, Japan) at 37°C for 2 hours.[2] Plates were rinsed, blocked with 1% FBS in PBS, incubated with 1:100 HI plasma diluted in 0.05% Tween-20/ 0.1% FBS in PBS for 2 hours then rinsed again, then incubated with IgG-HRP (1:5000, G8-185; BD). HRP was revealed by stabilized hydrogen peroxide and tetramethylbenzidine (R&D systems) for 20 minutes, stopped with 2 M H_2SO_4 and analyzed on an absorbance microplate reader at 450 nm wavelength (Tecan Life Sciences).

References:

1. Imamura, T., Isozumi, N., Higashimura, Y., Ohki, S. & Mori, M. Production of ORF8 protein from SARS-CoV-2 using an inducible virus-mediated expression system in suspension-cultured tobacco BY-2 cells. *Plant Cell Rep* **40**, 433-436 (2021).
2. Hachim, A., *et al.* ORF8 and ORF3b antibodies are accurate serological markers of early and late SARS-CoV-2 infection. *Nat Immunol* **21**, 1293-1301 (2020).

Supplemental Table 1. Plasmid construct details

	Primers		
**Gene**	**Forward**	**Reverse**	**Sequence ID**
**Nsp1**	CTGAGCGGATCCATGGAGAGCCTTGTCCCTG	CTGAGCGGCCGCCCCTCCGTTAAGCTCACG	QIA20043.1
**S1**	CTGACTCGAGATGTGTGTTAATCTT	CTGAGGTACCTGCCCGCCGAGGAGAATTA	QHD43416.1
**S2**	CTGACTCGAGATGCGTAGTGTAGCTAGTCAAT	CTGAGGTACCTGTGTAATGTAATTTGACTC	QHD43416.1
**S2'**	CTGACTCGAGATGAGGTCATTTATTGAAGA	CTGAGGTACCTGTGTAATGTAATTTGACTC	QHD43416.1
**N**	CTGAGCGGATCCATGTCTGATAATGGACC	CTGAGCGGCCGCTTAGGCCTGAGTTGAGTCAG	QHD43423.2
**M**	CTGAGCGGATCCATGGCAGATTCCAACGGTACT	CTGAGCGGCCGCGCAAAGCAATATTGTCACTGCTAC	QHD43418.1
**E**	CTGAGCGGATCCTGTACTCATTCGTTTCGGAAGA	CTGAGCGGCCGCGACCAGAAGATCAGGAACTC	QHD43417.1
**ORF3a**	CTGAGCGGATCCATGGATTTGTTTATGAG	CTGAGCGGCCGCTTACAAAGGCACGCTAGTAGTC	QHD43419.1
**ORF3d**	CTGAGCGGATCCATGGCTTATTGTTGGCG	CTGAGCGGCCGCAGGCCAGCAGCAACGAG	PMID31987001
**ORF6**	CTGAGCGGATCCATGTTTCATCTCGTTG	CTGAGCGGCCGCTTAATCAATCTCCATTG	QHD43420.1
**ORF7a**	CTGAGCGGATCCATGAAAATTATTCTT	CTGAGCGGCCGCTCATTCTGTCTTTCTTT	QHD43421.1"
**ORF7b**	CTGAGCGGATCCATGATTGAACTTTCATT	CTGAGCGGCCGCTTAGGCGTGACAAGTTTC	QIA20050.1
**ORF8**	CTGAGCGGATCCATGAAATTTCTTGTTTTC	CTGAGCGGCCGCTTAGATGAAATCTAAAAC	QHD43422.1
**ORF10**	CTGAGCGGATCCATGGGCTATATAAACGT	CTGAGCGGCCGCCTATGTGAGATTAAAGT	QHI42199.1

Supplemental Table 2. Correlation between variables and Principal Components 1 and 2 (Dimension 1 and 2 of 14, represented in Fig. 3d-f).

Dimension	Variable	Correlation	P value
1	N	0.5921686	0.00000
1	S1	0.0906619	0.10884
1	S2’	0.1981899	0.00041
1	S2	0.4552057	0.00000
1	ORF7a	0.5495707	0.00000
1	ORF3d	0.6786038	0.00000
1	ORF7b	-0.0681466	0.22853
1	NSP1	0.6656403	0.00000
1	ORF10	-0.2925584	0.00000
1	E	0.275052	0.00000
1	M	-0.1509175	0.00739
1	ORF6	-0.3774847	0.00000
1	ORF8	0.6342754	0.00000
1	ORF3a	0.6259408	0.00000
2	N	-0.0821349	0.14648
2	S1	0.6981859	0.00000
2	S2’	0.131899	0.01938
2	S2	0.3052223	0.00000
2	ORF7a	0.5271245	0.00000
2	ORF3d	0.3465305	0.00000
2	ORF7b	0.5554244	0.00000
2	NSP1	-0.1296366	0.02158
2	ORF10	0.608538	0.00000
2	E	-0.44982	0.00000
2	M	0.3325727	0.00000
2	ORF6	0.2425214	0.00001
2	ORF8	-0.1286999	0.02255
2	ORF3a	-0.0883815	0.11807

NB: Correlation between variables and Principal Components 1 and 2 (Dimension 1 and 2 of 14, represented in Fig. 3d-f). Testing of the correlations based on student's *t* distribution for Pearson's product moment correlation coefficients. The *t* test is two-sided and no adjustment was made for multiple comparisons.

Supplemental Table 3. Percentages of single antibody responses in asymptomatic/mild pediatric and adult populations (from Fig. 2j).

	Antigen	Pediatric (observed) %	Adult (expected) %
Structural	**S1**	4.83	6.52
	**S2**	3.41	4.03
	**S2'**	7.14	6.84
	**M**	8.06	9.88
	**E**	10.99	4.85
Accessory	**NSP1**	7.72	7.84
	**ORF3a**	12.66	14.71
	**ORF3d**	7.48	8.17
	**ORF6**	4.71	4.21
	**ORF7a**	5.62	6.60
	**ORF7b**	8.53	11.2
	**ORF8**	13.39	9.21
	**ORF10**	5.469	5.93

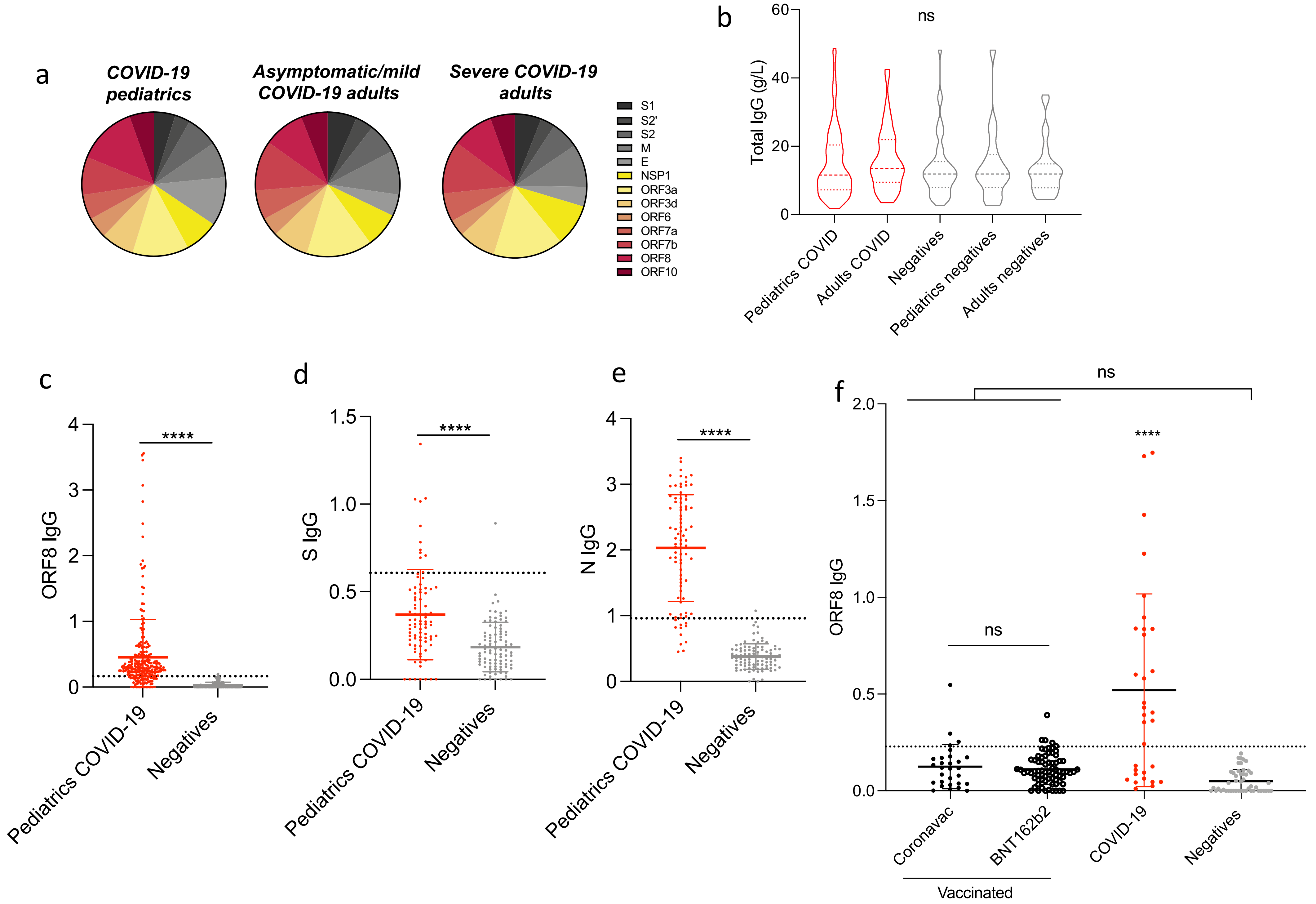
a
COVID-19 pediatrics
Asymptomatic/mild COVID-19 adults
Severe COVID-19 adults
S1
S2'
S2
M
E
NSP1
ORF3a
ORF3d
ORF6
ORF7a
ORF7b
ORF8
ORF10
b
Total IgG (g/L)
ns
Pediatrics COVID
Adults COVID
Negatives
Pediatrics negatives
Adults negatives
c
ORF8 IgG
****
Pediatrics COVID-19
Negatives
d
S IgG
****
Pediatrics COVID-19
Negatives
e
N IgG
****
Pediatrics COVID-19
Negatives
f
ORF8 IgG
ns
ns
****
Coronavac
BNT162b2
COVID-19
Negatives
Vaccinated

Supp Fig. 1

Supplemental figure 1. (**a**) Pie-charts representing the percentages of single antibody levels to SARS-CoV-2 antigens of the cumulative SARS-CoV-2 antibody response in COVID-19 pediatric cases, asymptomatic/mild adult cases and severe adult cases, for the antigen panel excluding N (13 antigens). (**b**) Levels of total IgG (g/L) in plasma measured by ELISA in the pediatrics COVID (n=247), adults COVID (n=44), negatives (n=45, pediatrics negatives (n=17), adults negatives (n=28)). (**c-e**) Antibodies against the SARS-CoV-2 proteins ORF8 (**c**, COVID-19 n= 243 and negatives n=184), S (**d**) and N (**e**) (COVID-19 n= 84 and negatives n=100) respectively. (f) Antibodies against the SARS-CoV-2 proteins ORF8 for Coronvac (inactivated whole virion) vaccinated (n=29), BNT162b2 (Spike mRNA lipoprotein) vaccinated (n=73), COVID-19 positives (n=32) and negatives (n=48). (**b-f**) represents individual IgG responses and the mean ± stdev. Statistical significance was established using a One-way ANOVA (**b, f**) or a student t-test (**c, d, e**). * shows statistical significance between COVID-19 patients versus negative controls. P values for (**c-e**) for Pediatrics COVID-19 versus Negatives are all p<0.0001 ****. P values for (**f**) are: for CoronaVac versus COVID-19: p<0.0001, BNT162b2 versus COVID-19: p<0.0001 ****, and negatives versus COVID-19: p<0.0001 ****. not significant = ns for Coronavac versus BNT162b2, negatives versus CoronaVac and BNT162b2

a

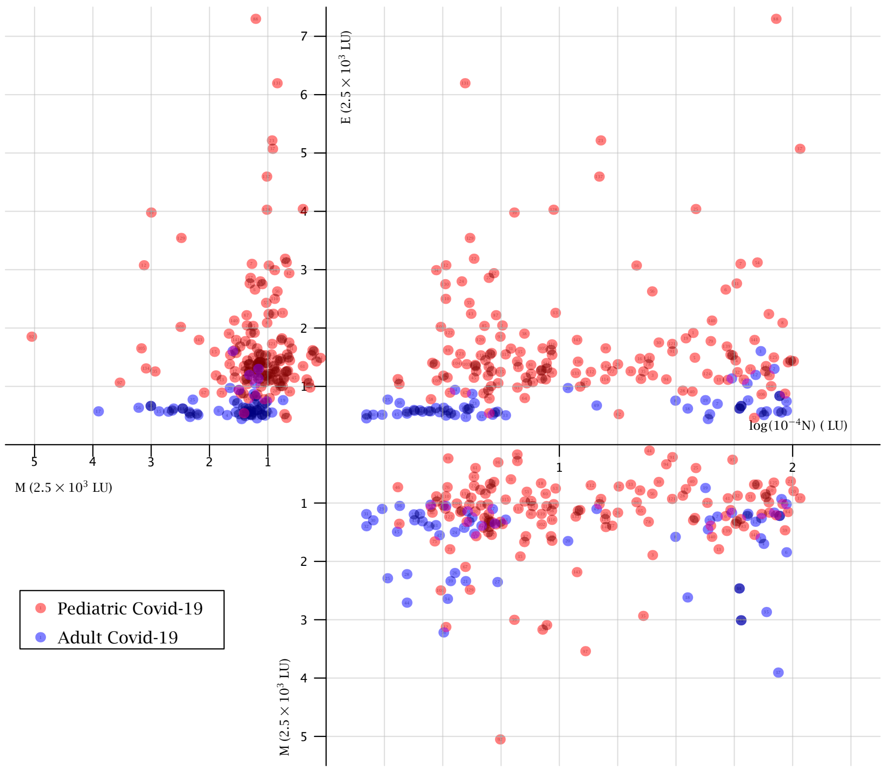
E (2.5 × 10³ LU)
log(10⁻⁴N) (LU)
M (2.5 × 10³ LU)
M (2.5 × 10³ LU)
Pediatric Covid-19
Adult Covid-19

b

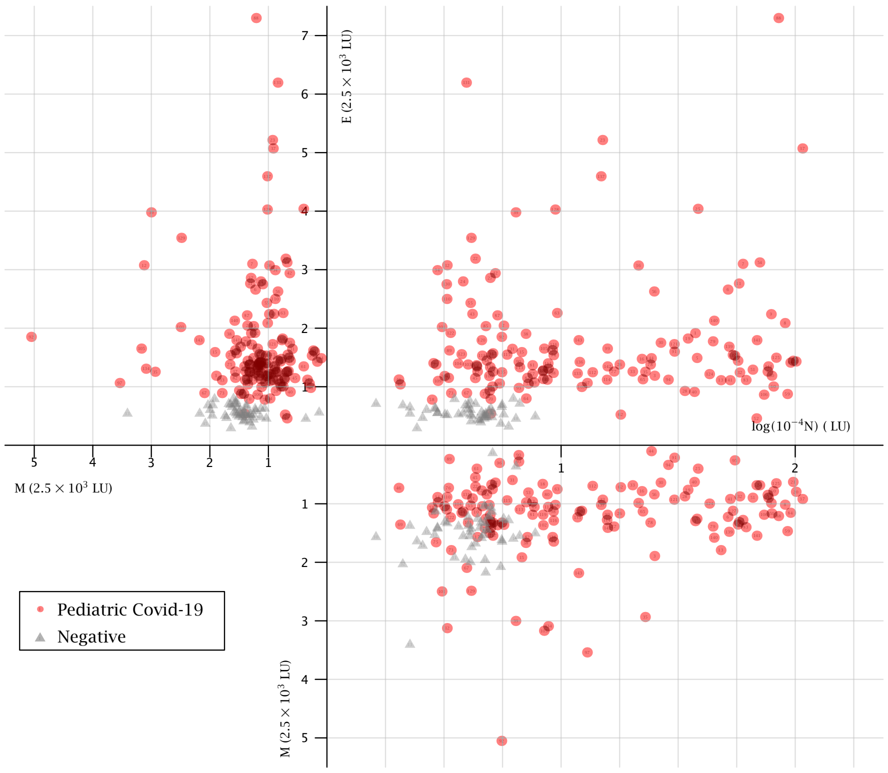
E (2.5 × 10³ LU)
log(10⁻⁴N) (LU)
M (2.5 × 10³ LU)
M (2.5 × 10³ LU)
Pediatric Covid-19
Negative

Supplemental Figure 2. Representation of the N, E, M response of pediatric COVID-19 population as clusters of points. Patients are presented according to their values of SARS-CoV-2 individual antibodies as (x, y, z) in the space. (**a**) N, M, E antibodies combination for the pediatric COVID-19 population versus the adult COVID-19 population (**b**) and for the pediatric COVID-19 population versus the negative population. COVID-19 children patients (n=144) are represented as red dots. COVID-19 adult patients (n=71, except for (**b**) where n=61) are represented as blue dots. The negative population (n=28) is represented as gray triangles.

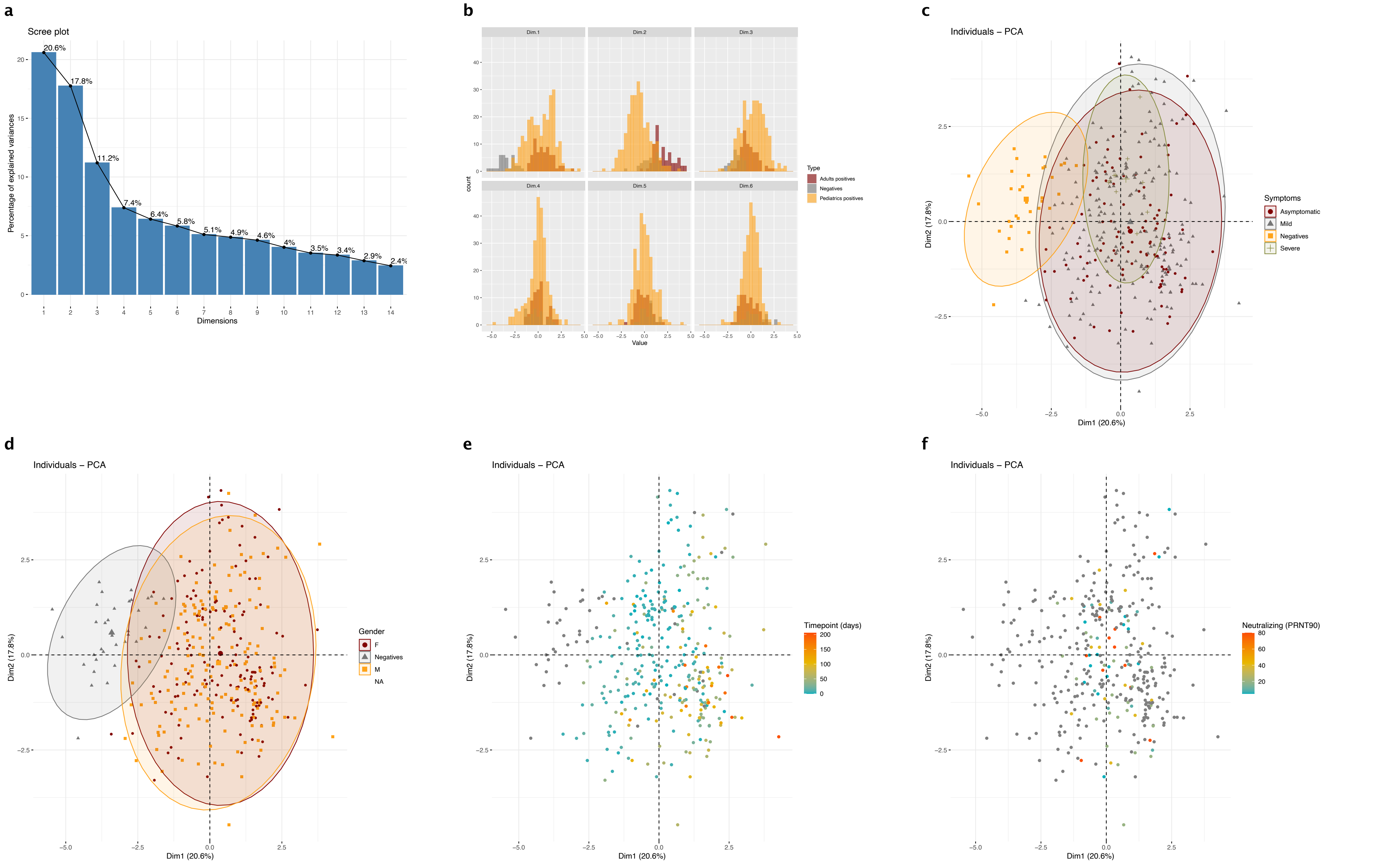

a
Scree plot
Percentage of explained variances
Dimensions
20.6%
17.8%
11.2%
7.4%
6.4%
5.8%
5.1%
4.9%
4.6%
4%
3.5%
3.4%
2.9%
2.4%
b
Dim.1
Dim.2
Dim.3
Dim.4
Dim.5
Dim.6
count
Value
Type
Adults positives
Negatives
Pediatrics positives
c
Individuals – PCA
Dim1 (20.6%)
Dim2 (17.8%)
Symptoms
Asymptomatic
Mild
Negatives
Severe
d
Individuals – PCA
Dim1 (20.6%)
Dim2 (17.8%)
Gender
F
Negatives
M
NA
e
Individuals – PCA
Dim1 (20.6%)
Dim2 (17.8%)
Timepoint (days)
f
Individuals – PCA
Dim1 (20.6%)
Dim2 (17.8%)
Neutralizing (PRNT90)

Supplemental Figure 3. Principal Component Analysis. (**a**) Scree-plot for explained variances of the principal component analysis. Dim1 and 2 explained 20.6 and 17.8% of the variances. (**b**) Distributions of the Principal Components (Dim1 and Dim2) colored by sample-type: Pediatric positives, Adult positives, Negatives. (**c-f**) Factorial plot of PCA on dimension 1 and 2 for symptoms (**b**), gender (**c**), time-point (**d**), symptoms (**e**) and neutralization data (PRNT90) (**f**).

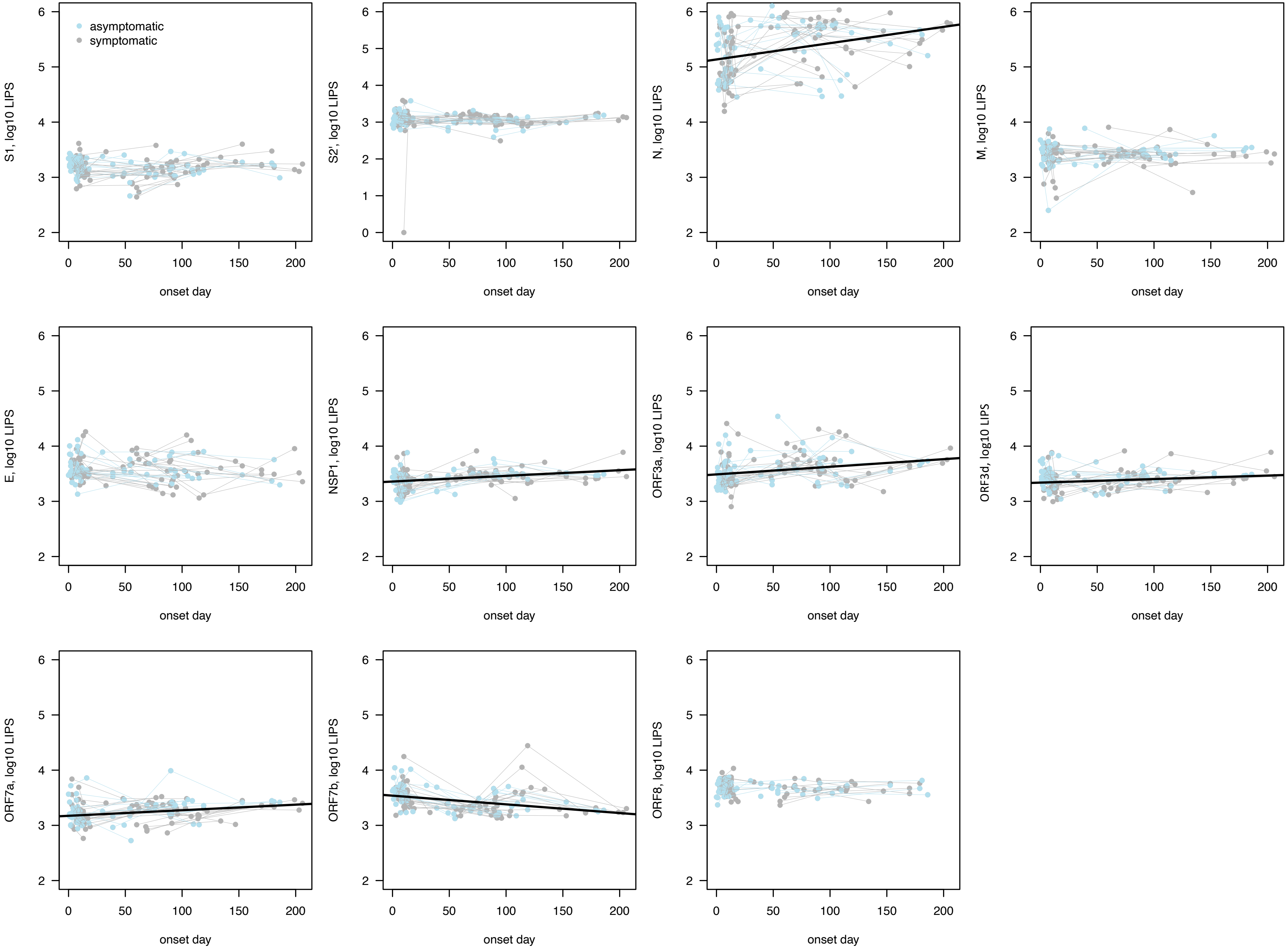

Supplemental Figure 4. Longitudinal antibody responses in asymptomatic and symptomatic COVID-19 children. Longitudinal analysis of 11 relevant antibody responses for structural (S1, S2', N, M, E) and non-structural (NSP-1, ORF3a, ORF3b, ORF7a, ORF7b, ORF8) SARS-CoV-2 proteins in asymptomatic (light blue) and symptomatic (grey) COVID-19 children.

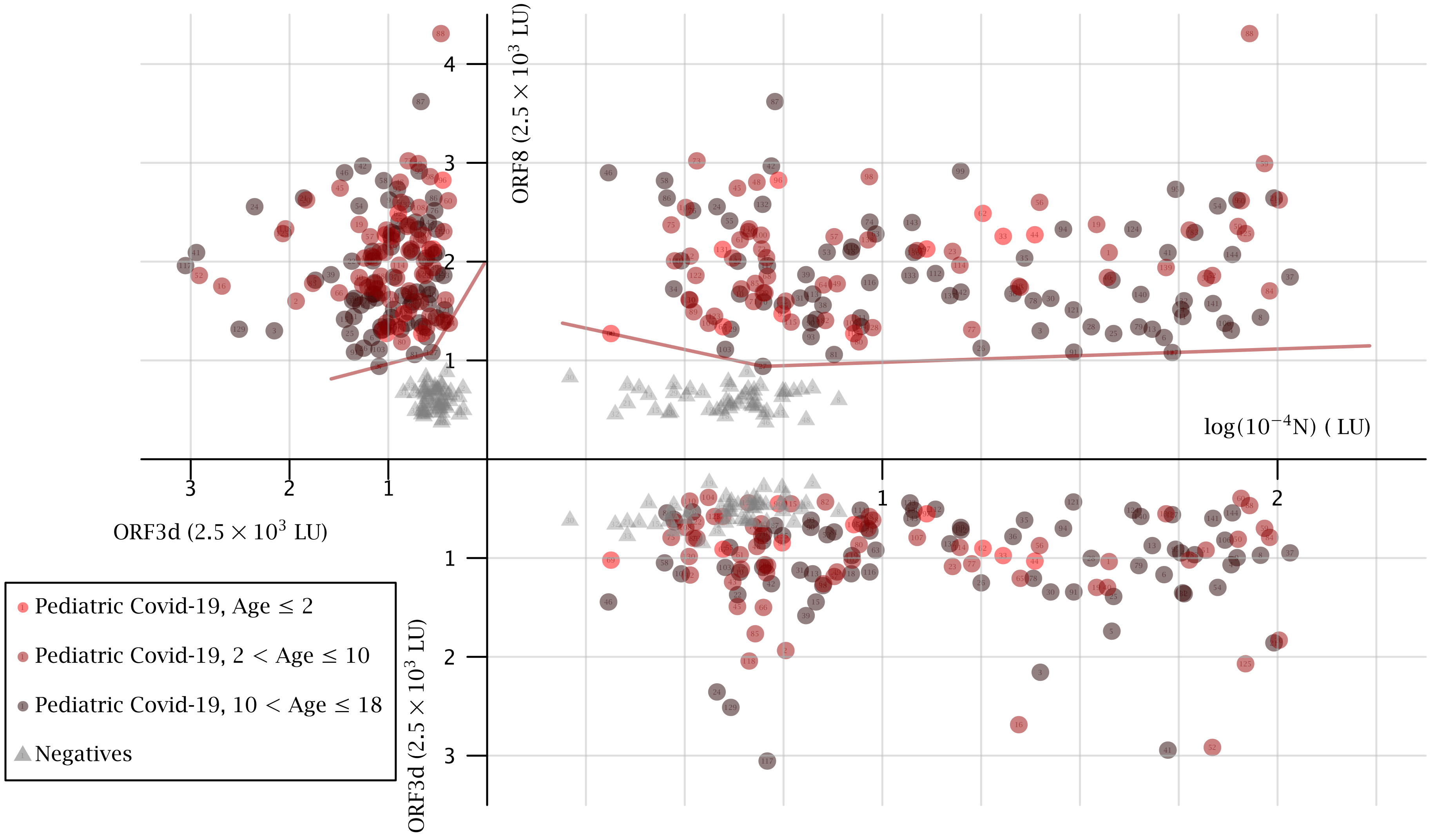
ORF8 (2.5 × 10^3 LU)
ORF3d (2.5 × 10^3 LU)
log(10^−4N) (LU)
Pediatric Covid-19, Age ≤ 2
Pediatric Covid-19, 2 < Age ≤ 10
Pediatric Covid-19, 10 < Age ≤ 18
Negatives

Supplemental Figure 5: Representation of the N ORF3d ORF8 response of pediatric COVID-19 population by age as clusters of points. Patients are presented according to their values of SARS-CoV-2 individual antibodies as $(x,\ y,\ z)$ in the space. Cluster for N, ORF8, ORF3d antibodies according to the children's age. For the red dots are darker according to the patient's age (0-2, 2-10, 11-18). COVID-19 children patients (n=144) are represented as red dots. The negative population (n=28) is represented as gray triangles.
