# Supplementary material for: SARS-CoV-2 accessory proteins reveal distinct serological signatures in children
Source: Nat Commun. 2022 May 26;13:2951. doi: 10.1038/s41467-022-30699-5 (PMC9135746; doi:10.1038/s41467-022-30699-5)
Supplement: Supplementary file 3 — Reporting Summary [file 41467_2022_30699_MOESM3_ESM.pdf]

nature portfolio

Corresponding author(s): Sophie Valkenburg

Last updated by author(s): 2022/04/21

Reporting Summary

Nature Portfolio wishes to improve the reproducibility of the work that we publish. This form provides structure for consistency and transparency in reporting. For further information on Nature Portfolio policies, see our Editorial Policies and the Editorial Policy Checklist.

Statistics

For all statistical analyses, confirm that the following items are present in the figure legend, table legend, main text, or Methods section.

n/a	Confirmed	
☐	☒	The exact sample size (*n*) for each experimental group/condition, given as a discrete number and unit of measurement
☒	☐	A statement on whether measurements were taken from distinct samples or whether the same sample was measured repeatedly
☐	☒	The statistical test(s) used AND whether they are one- or two-sided<br>*Only common tests should be described solely by name; describe more complex techniques in the Methods section.*
☐	☒	A description of all covariates tested
☒	☐	A description of any assumptions or corrections, such as tests of normality and adjustment for multiple comparisons
☐	☒	A full description of the statistical parameters including central tendency (e.g. means) or other basic estimates (e.g. regression coefficient) AND variation (e.g. standard deviation) or associated estimates of uncertainty (e.g. confidence intervals)
☐	☒	For null hypothesis testing, the test statistic (e.g. *F*, *t*, *r*) with confidence intervals, effect sizes, degrees of freedom and *P* value noted<br>*Give P values as exact values whenever suitable.*
☒	☐	For Bayesian analysis, information on the choice of priors and Markov chain Monte Carlo settings
☒	☐	For hierarchical and complex designs, identification of the appropriate level for tests and full reporting of outcomes
☒	☐	Estimates of effect sizes (e.g. Cohen's *d*, Pearson's *r*), indicating how they were calculated

Our web collection on statistics for biologists contains articles on many of the points above.

Software and code

Policy information about availability of computer code

Data collection	ELISA and LIPS samples were quantified using a Wallac MicroBeta JET luminometer 1450 LSC & Luminescence counter and Magellan3 and GLOMAX v1.1.7 software respectively for analysis (Promega), and the cytokine bead array acquired by Flow cytometry on an Invitrogen Attune according to manufacturers instructions.
Data analysis	Software used for data analysis include Prism (v10), Wallac MicroBeta JET luminometer 1450 LSC & Luminescence counter and GLOMAX v1.7.1 software for analysis (Promega). The cytokine bead array was analyzed with LegendPlex software (batch specific dongle).<br>The SARS-CoV-2 antibodies dataset has been analysed through the free software ConTeXt, with LuaMetaTeXengine (version 2020.05.18) developed by Hans Hagen (http://www.pragma-ade.nl) which uses TeX, Metapost and Lua to obtain the 3D clusters of points shown in Figure 3abc, Figure 7c and Supplemental Figure 2 and 5.<br>The LU for 14 antigens were log-scale transformed (the negative and zero values in the data set were replaced by 1) prior to PCA analysis. The missing values in the dataset were estimated by a probabilistic model. The probabilistic model is tolerant to amounts of missing values between 10% to 15% which is fit for our data. The missing data was estimated using pcaMethods (version 1.80.0). The completed data were standardized (scaled) before input in standard PCA (using FactoMineR (version 2.4). The PCA results were extracted and visualized using factoextra (version 1.0.7)

For manuscripts utilizing custom algorithms or software that are central to the research but not yet described in published literature, software must be made available to editors and reviewers. We strongly encourage code deposition in a community repository (e.g. GitHub). See the Nature Portfolio guidelines for submitting code & software for further information.

Data

Policy information about availability of data

All manuscripts must include a data availability statement. This statement should provide the following information, where applicable:

- Accession codes, unique identifiers, or web links for publicly available datasets
- A description of any restrictions on data availability
- For clinical datasets or third party data, please ensure that the statement adheres to our policy

Data availability statement:
The raw data that support the findings of this study included as supplementary files, from which PCA analysis is derived from (Fig. 3d-h, and 7b-d). Data from LIPS and ELISA IgG responses with background subtracted are indicated in all figures are shown.

Field-specific reporting

Please select the one below that is the best fit for your research. If you are not sure, read the appropriate sections before making your selection.

☒ Life sciences ☐ Behavioural & social sciences ☐ Ecological, evolutionary & environmental sciences

For a reference copy of the document with all sections, see nature.com/documents/nr-reporting-summary-flat.pdf

Life sciences study design

All studies must disclose on these points even when the disclosure is negative.

Sample size	This is an opportunistic study that determined the sample size. We used n=122 pediatric patients, with heterogeneity in terms of age and symptomatology (symptomatic n=78, asymptomatic n=44). These pediatric samples were compared to 71 infected adults (symptomatic n=61, and asymptomatic n=10), and negative controls (n=48).
Data exclusions	No data were excluded
Replication	The reproducibility of the LIPS method was assessed in previous studies (Hachim A et al. Nat Immunol. 2020 Oct;21(10):1293-1301.)<br>The CBA was run twice with samples run in duplicates each time: the first CBA experiment was not valid due to standards and controls being out of range, and therefore a second experiment was run with a new CBA kit that did not show any technical issue. Results from the first experiment were not used in the study.
Randomization	Not relevant to our observational study.
Blinding	We were not blinded to subjects as recruitment was based on RT-PCR confirmed COVID-19 infection, and negative controls served as assay controls.

Reporting for specific materials, systems and methods

We require information from authors about some types of materials, experimental systems and methods used in many studies. Here, indicate whether each material, system or method listed is relevant to your study. If you are not sure if a list item applies to your research, read the appropriate section before selecting a response.

Materials & experimental systems

n/a	Involved in the study	
☐	☒	Antibodies
☐	☒	Eukaryotic cell lines
☒	☐	Palaeontology and archaeology
☒	☐	Animals and other organisms
☐	☒	Human research participants
☒	☐	Clinical data
☐	☐	Dual use research of concern

Methods

n/a	Involved in the study	
☒	☐	ChIP-seq
☒	☐	Flow cytometry
☒	☐	MRI-based neuroimaging

Antibodies

Antibodies used	Secondary ELISA antibody: IgG-HRP (1:5,000, clone: G8-185; BD, cat# 55578)
Validation	Titration and used across vaccination studies (see PMID: 33594050).

Eukaryotic cell lines

Policy information about cell lines

Cell line source(s)	Cos1 cells from American Type Culture Collection (ATCC)
Authentication	Morphology check by microscope, only cells at early passage have been used.
Mycoplasma contamination	Mycoplasma report from ATCC and low passage used. In-house mycoplasma testing was not performed as only low passage cells were used.
Commonly misidentified lines (See ICLAC register)	none

Human research participants

Policy information about studies involving human research participants

Population characteristics	The age and gender of patients and controls is described in Table 1.
Recruitment	Participants were recruited based on available patients with RT-PCR confirmed COVID-19 infection in Hong Kong and in the USA. All participants provided informed consent or assent. Their blood was collected at various time-point after disease onset, and there was no bias to the recruitment or collection. As participation was voluntary, circulation of SARS-CoV-2 was limited during the study period in Hong Kong leading to small infection waves in 2020, most cases were mild or asymptomatic in Hong Kong, our cohort may have self selection bias to mild COVID-19 as described in table 1 and severe cases were included from the USA due to medical care of these patients and the scale of the pandemic in the USA in 2020. For transparency data is presented according to symptom severity and age groups defined.
Ethics oversight	The COVID-19 patient study was approved by the institutional review board of the respective hospitals, viz. Kowloon West Cluster (KW/EX-20-039 (144-27)), Kowloon Central / Kowloon East cluster (KC/KE-20-0154/ER2) and HKU/HA Hong Kong West Cluster (UW 20-273, UW20-169), Joint Chinese University of Hong Kong-New Territories East Cluster Clinical Research Ethics Committee (CREC 2020.229), and the Human Research Protection Office at Washington University in St. Louis, USA (IRB reference number 202007097). The collection of negative control blood donors was approved by the Institutional Review Board of The Hong Kong University and the Hong Kong Island West Cluster of Hospitals (approval number: UW16-254).

Note that full information on the approval of the study protocol must also be provided in the manuscript.

Dual use research of concern

Policy information about dual use research of concern

Hazards

Could the accidental, deliberate or reckless misuse of agents or technologies generated in the work, or the application of information presented in the manuscript, pose a threat to:

No	Yes	
☒	☐	Public health
☒	☐	National security
☒	☐	Crops and/or livestock
☒	☐	Ecosystems
☒	☐	Any other significant area

Experiments of concern

Does the work involve any of these experiments of concern:

No	Yes	
☒	☐	Demonstrate how to render a vaccine ineffective
☒	☐	Confer resistance to therapeutically useful antibiotics or antiviral agents
☒	☐	Enhance the virulence of a pathogen or render a nonpathogen virulent
☒	☐	Increase transmissibility of a pathogen
☒	☐	Alter the host range of a pathogen
☒	☐	Enable evasion of diagnostic/detection modalities
☒	☐	Enable the weaponization of a biological agent or toxin
☒	☐	Any other potentially harmful combination of experiments and agents
