# Supplementary material for: SARS-CoV-2 accessory proteins reveal distinct serological signatures in children
Source: Nat Commun. 2022 May 26;13:2951. doi: 10.1038/s41467-022-30699-5 (PMC9135746; doi:10.1038/s41467-022-30699-5)
Supplement: Supplementary file 2 — Peer Review File [file 41467_2022_30699_MOESM2_ESM.pdf]

nature portfolio

Peer Review File

SARS-CoV-2 accessory proteins reveal distinct serological signatures in children

Open Access This file is licensed under a Creative Commons Attribution 4.0 International License, which permits use, sharing, adaptation, distribution and reproduction in any medium or format, as long as you give appropriate credit to the original author(s) and the source, provide a link to the Creative Commons license, and indicate if changes were made. In the cases where the authors are anonymous, such as is the case for the reports of anonymous peer reviewers, author attribution should be to 'Anonymous Referee' followed by a clear attribution to the source work. The images or other third party material in this file are included in the article's Creative Commons license, unless indicated otherwise in a credit line to the material. If material is not included in the article's Creative Commons license and your intended use is not permitted by statutory regulation or exceeds the permitted use, you will need to obtain permission directly from the copyright holder. To view a copy of this license, visit http://creativecommons.org/licenses/by/4.0/.

Reviewers' Comments:

Reviewer #1:
Remarks to the Author:
The relevance of the study by Hachim and colleagues lies on (i) the focus on serological responses to less commonly studied structural and non-structural SARS-CoV-2 antigens (beyond S and N) that could be good targets for diagnosis (including vaccine breakthroughs) and understanding immunity to COVID with a wider immunological breadh (ii) includes children who have been less studied and compares them to adults, trying to classify signatures that can discriminate (iii) includes asymptomatic children that is a more rare population to detect, and (iv) attempts to relate the antibody to cytokine profiles. The most noteworthy results are the detailed signatures that distinguish seroreactivities by age. However they are not translated into symptomatology or protection.

The work can be significant in the field of diagnostics and more so after vaccination.

However, the work does not totally support conclusions (title) and further evidence is needed from prospective studies. Specifically, it claims that the work revealed potential protective antigen targets but it is not clear in the paper or abstract how protection is defined and demonstrated from the data, particularly if no difference by symptoms was detected, and beyond correlation analysis with cytokines involved in severity.

There are some limitations in the data preprocessing and analysis that require substantial revision as specified below. The methodology requires also more detail as it is not standard, and further information required in the paper to allow reproducibility. Data are provided upon request.

Specific requird reviews:

ABSTRACT
- Basic information lacking: Provide the location and number of children and adults analysed, for each symptoms category, what antibody isotype and what immunoassay
- It is descriptive and lacks a conclusion and biomedical implication of results

INTRODUCTION
- Among the hypotheses for lower morbidity in children, there is no reference to the different expression of ACE2
- Some of the references are preprints, recheck if they have been published in peer-review journals
- A differential serological reactivity to S in children compared to adults is mentioned, but no reference to N that has also shown differences by age, please review and comment too
- It is not clear what is meant by "landscape", please clarify or use another more commonly used term
- Define LIPS (not a common term or technique)

METHODS
- From Table 1 and the text, it is not clear how the samples were collected in relation to individuals. It is understood that multiple samples came from each or some person but more detail is required in terms of what timepoints in respect to infection and symptoms. A flowchart or a schematic graphic will help for this. Not clear how repetitive measures per individual are considered in the dotploy analyses
- For the panel of 14 proteins (S1, S2, S2’, E, M, N, NSP1, ORF3a, 3b, 6, 7a, 7b, 8, 10), beyond giving a reference, provide here more details for the readers of this paper in relation of whether they were full lenght of fragments, and which ones (e.g. if RBD contained). Indicate the meaning of comma: S2’
- More background information on the Ruc and LIPS are required since they are not standard methodologies. E.g. if they are similar to a peptide/protein array (slide or bead based) or other technology, as well as performance (sensitivity and specificity in validation studies with sufficient sample numbers)
- State if authors tested effect of heat inactivation on antibody levels and outcome

- The "cluster of points" paragraph and subsequents: redefine to make clearer these refer to data analysis
- Use the term sex rather than gender for biological effect

RESULTS
- Indicate everywhere what antibodies are measured, is it IgG? The study would be richer if IgM and IgA would be included as they are also relevant for the objectives
- A major issue is with the way data are presented. Negative prepandemic controls are routinely used to establish seropositivity thresholds but here the cutoffs are not shown.
- The number of controls is limited (33 only, and even less, 20, for children).
- The plots compare levels of negative controls to pandemic samples that in many cases are below the prepandemic concentration median, therefore are negative; then, what is the meaning of comparing negative to negative? The analysis should be based on existing antibody responses (ie. positive).
- If there is concern that cutoff would vary in children vs adults due to differnt background (e.g. cross reactivity with CCoVs) then it should be stratified and with higher numbers. This is usually more of a concern with N and S2 than to S1. Actually, adult and children negative plasmas could be marked in different symbols in the graphs to see if they overlap

DISCUSSION
- As mentioned above, the main strengh is the potential of the markers to distinguish adults from children serologically, in principle for diagnostics purpose and seroprevalence studies, but this is not well linked to protection, therefore the title need to be changed
- There are some references that are lacking on large antibody-based studies to understand the different dynamics of SARS-CoV-2 transmission in children vs adults, including detection in plasma/serum surveys in different countries, and also in saliva, using S and N proteins. Please review and update
- This is probably one of the more relevant features that needs to be more highlighted: "the interest in antibodies to SARS-CoV-2 internal proteins will grow with the rollout of sub-unit Spike only vaccines, in order to allow the distinction between SARS-CoV-2 past infection and vaccination in specific populations and to create an estimated date of exposure given the unique rate of waning of different specificities". Furthermore when there are evidences that antibodies to N may not be as useful for this purpose (reference lacking for this).

Reviewer #2:
Remarks to the Author:
Comments
COVID-19 is still unfolding. It is known that after infection the severity and morbidity is significantly different between children and adult. However, the underlying mechanism and the difference of global antibody responses are largely unknown. In this study, the authors compared the antibody responses against 14 SARS-CoV-2 proteins, also analyzed a panel of immune priming and inflammation related cytokines. They observed differences of the antibody responses and cytokine levels and also purposed the possible applications of these observed differences. This is basically an observational study, the findings may broaden our understanding of the immune response against COVID-19, especially for Children.

Major concerns:
1. This study is basically observational. To make their conclusions more convincing, the findings need to be validated at least with a small cohort of independent samples, better using an approach other than LIPS.
2. There are a variety of publications about the COVID-19 related antibody responses, for both the structural and non-structural proteins, for example, the protein microarray related studies. These studies should be properly cited, a comprehensive comparison should be performed between this

study and the previous related studies.
3. Longitudinal samples were collected. It's better to perform some longitudinal analysis for these samples.

Minor concerns:
1. What is "LIPS"? The full name and some explanation is needed for the first appearance.
2. It is not correct to state "There is a lack of information on the SARS-CoV-2 antibody responses in adults or children to the virus accessory proteins which can modulate immune responses", it is not the case at least for adults.
3. "whereas no difference was observed for S2 antibodies", please explain this.
4. "we excluded the early time-point samples (<14 days of infection) from the adult cohort", what is rationale for this?

REVIEWER COMMENTS
Reviewer #1 (Remarks to the Author):

The relevance of the study by Hachim and colleagues lies on (i) the focus on serological responses to less commonly studied structural and non-structural SARS-CoV-2 antigens (beyond S and N) that could be good targets for diagnosis (including vaccine breakthroughs) and understanding immunity to COVID with a wider immunological breadth (ii) includes children who have been less studied and compares them to adults, trying to classify signatures that can discriminate (iii) includes asymptomatic children that is a more rare population to detect, and (iv) attempts to relate the antibody to cytokine profiles. The most noteworthy results are the detailed signatures that distinguish seroreactivities by age. However they are not translated into symptomatology or protection.

The work can be significant in the field of diagnostics and more so after vaccination.

However, the work does not totally support conclusions (title) and further evidence is needed from prospective studies. Specifically, it claims that the work revealed potential protective antigen targets but it is not clear in the paper or abstract how protection is defined and demonstrated from the data, particularly if no difference by symptoms was detected, and beyond correlation analysis with cytokines involved in severity.

There are some limitations in the data preprocessing and analysis that require substantial revision as specified below. The methodology requires also more detail as it is not standard, and further information required in the paper to allow reproducibility. Data are provided upon request.

Specific required reviews:

ABSTRACT
R1.1 - Basic information lacking: Provide the location and number of children and adults analysed, for each symptoms category, what antibody isotype and what immunoassay
- It is descriptive and lacks a conclusion and biomedical implication of results

A1.1 We thank the reviewer for their comments.

- We have improved the abstract by providing information on the number of subjects included in the study, with symptoms categories, antibody isotype covered by our assays, and detailed name of the immunoassay.
- We have added a new sentence for conclusion and biomedical implication of the results

  The new abstract with changes highlighted is included below:

  ABSTRACT:

The antibody response magnitude and kinetics may impact clinical severity, serological diagnosis and long-term protection of COVID-19, and children experience lower morbidity. We tested samples from 122 children in Hong Kong with symptomatic (n=78) and asymptomatic (n=44) SARS-CoV-2 infections up to 200 days post infection, relative to 71 infected adults (symptomatic n=61, and asymptomatic n=10), and negative controls (n=48). We assessed serum IgG antibodies to a 14-wide antigen panel of structural and accessory

proteins by Luciferase Immuno-Precipitation System (LIPS) assay and circulating cytokines. Infected children have lower levels of Spike, Membrane, ORF3a, 7a, 7b antibodies, comparable ORF8 and elevated E-specific antibodies than adults. Combination of two unique antibody targets, ORF3d and ORF8, can accurately discriminate SARS-CoV-2 infection in children. Principal component analysis revealed distinct pediatric serological signatures, and the highest contribution to variance from adults were responses to non-structural proteins ORF3d, NSP1, ORF3a and ORF8. From a diverse panel of cytokines that can modulate immune priming and relative inflammation, the chemokines IL-8, MCP-1 and IL-6 correlated with the magnitude of paediatric antibody specificity and severity. Antibodies to SARS-CoV-2 internal proteins may become an important sero surveillance tool of infection with the roll-out of vaccines in the pediatric population.

INTRODUCTION

R1.2 - Among the hypotheses for lower morbidity in children, there is no reference to the different expression of ACE2

A1.2 Apologies for our oversight, ACE2 expression is lower in children, we have added the study by Bunyavanich, et al in JAMA 2020 (doi:10.1001/jama.2020.8707) to the introduction.

R1.3 - Some of the references are preprints, recheck if they have been published in peer-review journals

A1.3 – We have checked and updated pre-prints that have moved to peer reviewed publications during the preparation of our manuscript, and updated some pre-prints to other related papers. No pre-print remain in our references.

R1.4 - A differential serological reactivity to S in children compared to adults is mentioned, but no reference to N that has also shown differences by age, please review and comment too

A1.4 We thank the reviewer for pointing out this missing reference. The following sentences have been added to the introduction (Page 4, line 22):

"N-specific antibody waning is more pronounced than S (10.1016/j.jinf.2021.03.015) and children are more likely to test S-antibody positive even in the absence of vaccination (doi.org/10.1016/j.jinf.2021.11.019). Therefore, N-based serology in children is also limited."

R1.5 - It is not clear what is meant by "landscape", please clarify or use another more commonly used term

A1.5 - As we have quantified the antibody response to a number of SARS-CoV-2 antigens, the landscape refers to a multi protein antibody response, i.e. the antibody proteome landscape. We have updated the text from 'landscape' to 'response' or 'specificity' for clarity instead.

R1.6 - Define LIPS (not a common term or technique)

A1.6 - The Luciferase Immunoprecipitation System (LIPS) is now defined in the abstract and given in the list of abbreviations. The methods section has also been expanded to describe the technique.

METHODS

R1.7 - From Table 1 and the text, it is not clear how the samples were collected in relation to individuals. It is understood that multiple samples came from each or some person but more detail is required in terms of what timepoints in respect to infection

and symptoms. A flowchart or a schematic graphic will help for this. Not clear how repetitive measures per individual are considered in the dotplot analyses
A1.7 - Our RT-PCR confirmed SARS-CoV-2 infected cohort is derived from an observational cohort study in Hong Kong. Patients were recruited with informed consent by their treating physician and sampled at hospital/quarantine admission and discharge, and followed up longitudinally when they next visited their doctor as part of the Hong Kong public health response to COVID-19. For adults this was typically at 3, 6 and 12 months post infection, and for children it was as they were available.
The sample numbers and time periods are shown in Figure 6ab to clarify the sampling details.

Our study enrolled a total of 122 pediatric patients, 108 of whom had only one blood-draw, and 58 of whom had 2 to 4 blood draws. Figure 6ab shows the details of longitudinal sampling:

- 58 pediatric subjects were sampled more than one time:
  - 35 had 2 blood draws
  - 20 had 3 blood draws
  - 3 had 4 blood draws

We added this information in a new version of Table 1.

The dot plots in Figures 1 and 2 consider the whole pediatric samples (total of 254 samples, including longitudinal samples), and Figure 6 depicts longitudinal samples only.
Repetitive measures per individual but different time-points are included in Figure 1 and 2.

R1.8 - For the panel of 14 proteins (S1, S2, S2', E, M, N, NSP1, ORF3a, 3b, 6, 7a, 7b, 8, 10), beyond giving a reference, provide here more details for the readers of this paper in relation of whether they were full length of fragments, and which ones (e.g. if RBD contained). Indicate the meaning of comma: S2'
A1.8 The sequences of the cloned regions are included in our original pre-print, cited in the methods based on the early genomic characterization fo SARS-CoV-2. The RBD is included in the S1, and S2' is the cleaved form the S2. This is described in the second paragraph of the first results section:
"We therefore first determined the levels of antibodies to different S sub-units by using 3 different S constructs in the LIPS assay: S1 which contains the RBD domain, S2 and the S2' cleaved subunit (**Fig. 1**)."
We also added a new table summarizing the plasmid construct details for each of the 14 proteins (new Supplemental Table 1) with protein accession codes for further sequence information.

R1.9 - More background information on the Ruc and LIPS are required since they are not standard methodologies. E.g. if they are similar to a peptide/protein array (slide or bead based) or other technology, as well as performance (sensitivity and specificity in validation studies with sufficient sample numbers)
A1.9 - We agree with the reviewer and have modified the paragraphs on the LIPS assays in the Materials and Methods section to more adequately describe our approach, as follows (page 22, line 22)

"**Luciferase Immunoprecipitation System (LIPS) assay**
The LIPS assay is a liquid phase immune-assay allowing the unbiased quantification of antibodies by measuring luminescence emitted by the reporter enzyme Renilla luciferase (Ruc) fused to an antigen of interest, expressed by the pRen2 vector in mammalian cells.

Luciferase, a light-producing enzyme is used as a reporter. The construction of *Renilla* luciferase (Ruc) chimeric genes involves the mammalian expression vectors pREN2 in which the antigen of interest is fused in-frame with Ruc. Protein A/G beads capture the immunoglobulin-antigen complexes. Protein A/G binds predominantly to the four isotypes of IgG but can also bind, to a much lesser extent, to IgA and IgM. Therefore, our data primarily represents plasma IgG. The use of LIPS in the diagnostic of SARS-CoV-2 infection has been published by us and others (for the 14 antigens tested in the present study [24], for Spike and Nucleocapsid [61-63]).
Based on previous studies describing the structure of the SARS-CoV-2 genome[48,64], an extensive panel of 14 proteins (S1, S2, S2', E, M, N, NSP1, ORF3a, 3, 6, 7a, 7b, 8, 10) was chosen for antibody testing by LIPS. Primers for the amplification of SARS-CoV-2 proteins were designed (See protein ID and primers sequences in Supplemental table 1). Primers and cloning for the amplification of SARS-CoV-2 proteins were as previously described[24]. Constructs with pREN2-Renilla luciferase plasmid containing the SARS-CoV-2 antigen of interest were transfected into Cos1 cells using Fugene 6 (Promega) as per manufacturer's instructions. Cells were harvested 48 hours later, lysed and sonicated, and (Ruc)-antigen yields were measured using a Luminometer plate reader (PerkinElmer) according to the protocol of Burbelo *et al.*(ref Burbelo) with the following modifications[59], as previously described[24].
Briefly, (Ruc)-antigen (at an equal concentration for each antigen at 10^7 per well) and plasma (heat inactivated and diluted 1:100) were incubated for 2 hours with shaking at 800rpm. Ultralink protein A/G beads (Thermo-Fisher) were added to the (Ruc)-antigen and serum mixture in a 96-deep-well polypropylene microtiter plate and incubated for 2 hours with shaking at 800rpm. The entire volume was then transferred into HTS plates (Millipore) and washed as previously described. The plate was read using QUANTI-Luc Gold substrate (Invivogen) as per manufacturer's instructions on a Wallac MicroBeta JET luminometer 1450 LSC & Luminescence counter and its software for analysis (PerkinElmer). Experimental controls include no plasma blank wells with (Ruc)-antigens and negative control serum from healthy donor plasmas collected prior to the COVID-19 pandemic (Table 1). The background corresponds to the LU signal from each Ruc-fusion antigen with protein A/G and substrate with no plasma. Responses were considered negative when they were not significantly elevated compared to negative pre-pandemic controls."

R1.10- State if authors tested effect of heat inactivation on antibody levels and outcome

A1.10- When we initially established the LIPS assay in our lab we tested non-heat inactivated samples however they showed higher background levels of binding. Therefore, we used heat inactivation going forward. Heat inactivation is a standard procedure for most serological studies, and has been tested by others (doi.org/10.1038/s41591-020-0913-5).
We have added the following statement on heat-inactivation in the Methods section (Page 22, line 19):

"All samples from COVID-19 patients or negative controls were heat-inactivated prior to experimental use at 56°C for 30 minutes (Heat-inactivated and non-heat inactivated samples were initially tested in the laboratory and by others (doi.org/10.1038/s41591-020-0913-5)."

R1.11 - The "cluster of points" paragraph and subsequents: redefine to make clearer these refer to data analysis

A1.11 -

We have modified the paragraph as follows:
“Analysis and representation of the dataset as clusters of points
The SARS-CoV-2 antibodies dataset has been analysed through the free software ConTeXt, with LuaMetaTeXengine (version 2020.05.18) developed by Hans Hagen (http://www.pragma-ade.nl) which uses TeX, Metapost and Lua to obtain the 3D clusters of points shown in Fig. 3abc, Fig. 7c and Supplemental Fig. 2. In the clusters of points, each sample is represented according to 3 parameters in the 3 axes (x, y, z). This analysis of the dataset considers a combination of three different parameters taken together. For clarity, only the first 144 COVID-19 pediatric samples of the dataset are represented in the clusters of points, along with the total COVID-19 adult samples (n=71) and negatives (n=48).
In the cluster (N, ORF3d, ORF8), the equations of the red lines are: (1) in the plane (N, ORF8) :830*log (N) +0.3843*ORF8=4801 and -350*log (N) +1.036*ORF8=790, and (2) in the plane (ORF3d, ORF8): 0.035*ORF3d+0.1334*ORF8=409.284 and 0.074*ORF3d+0.0437*ORF8=221.812. These straight lines allow the most accurate discrimination between negative controls and positive adult populations.”

R1.12 - Use the term sex rather than gender for biological effect
A1.12 Gender has been updated to sex in the text and Table 1.

RESULTS
R1.13 - Indicate everywhere what antibodies are measured, is it IgG? The study would be richer if IgM and IgA would be included as they are also relevant for the objectives
A1.13 - Protein A/G beads were used in the LIPS assay to precipitate immune complexes. Protein A/G binds predominantly to the four isotypes of IgG but can also bind, to a much lesser extent, to IgA and IgM. The LIPS technology does not allow for the measurement of the distinct levels of the different immunoglobulin classes/subclasses.
We agree with the reviewer that including data on IgM and IgA would be relevant to our study, and we trialed Protein L beads with technical limitations. ELISA for IgM as a secondary antibody would be possible if recombinant proteins were available for the ORFs of interest, this is a further area of study by our group which is beyond the scope of this study.

The specificity of Protein A/G beads has been added to the LIPS methods section (page 23, line 6):
“Protein A/G binds predominantly to the four isotypes of IgG but can also bind, to a much lesser extent, to IgA and IgM. Therefore, our data primarily represents serum IgG.”
We updated figure1 legend to include this description for subsequent figures.
Page 32, line 9
“All data represents individual time point IgG responses and to a lesser extent IgA and IgM based on protein A/G bead binding by LIPS and the mean ± stdev.”

Finally, a sentence has also been added to the Discussion section to highlight this limitation (page 21, line 4):
“Furthermore, the detection of various isotypes of antibodies for internal proteins such as IgM and IgA could also be relevant to assess the class switching, kinetic and Fc-related functions of the responses.”

R1.14 - A major issue is with the way data are presented. Negative prepandemic controls are routinely used to establish seropositivity thresholds but here the cutoffs are not shown.

A1.14 - We agree with the reviewer and have calculated the cut-offs as the mean ±3 SD of the negative population for each antibody test. Dotted lines to represent cut offs are now included in the graphs for the 14 antibody tested by LIPS in figures 1 and 2 and for the new graphs of the 3 antibody tested by ELISA in supplemental figure 1 c-f.

R1.15 - The number of controls is limited (33 only, and even less, 20, for children).
A1.15 - We agree with the reviewer that the number of controls is relatively low compared to sample number of COVID cases.
We were able to add 15 adult pre-pandemic controls to our initial control cohort and have now a total of 48 pre-pandemic controls.
In addition, we had previously validated and published the use of LIPS for these antigenic targets with a very large number of negative controls (n=191) and COVID-19 positive adults (n= 84) (Hachim and Kavian et al., Nat Imm 2020).
Regarding the number of negative pre-pandemic pediatric samples, we unfortunately were not able to access more samples with the required volume from our samples biobank, as access is limited in samples from healthy children. We included pre-pandemic plasma from 20 children to validate our findings, and the main comparisons in the present paper were between infected children and infected adults. Furthermore, we compared the detection of responses of the negative pediatric and negative adult populations and did not find any significant difference for any of the antigens tested (Reply Figure 1 below).

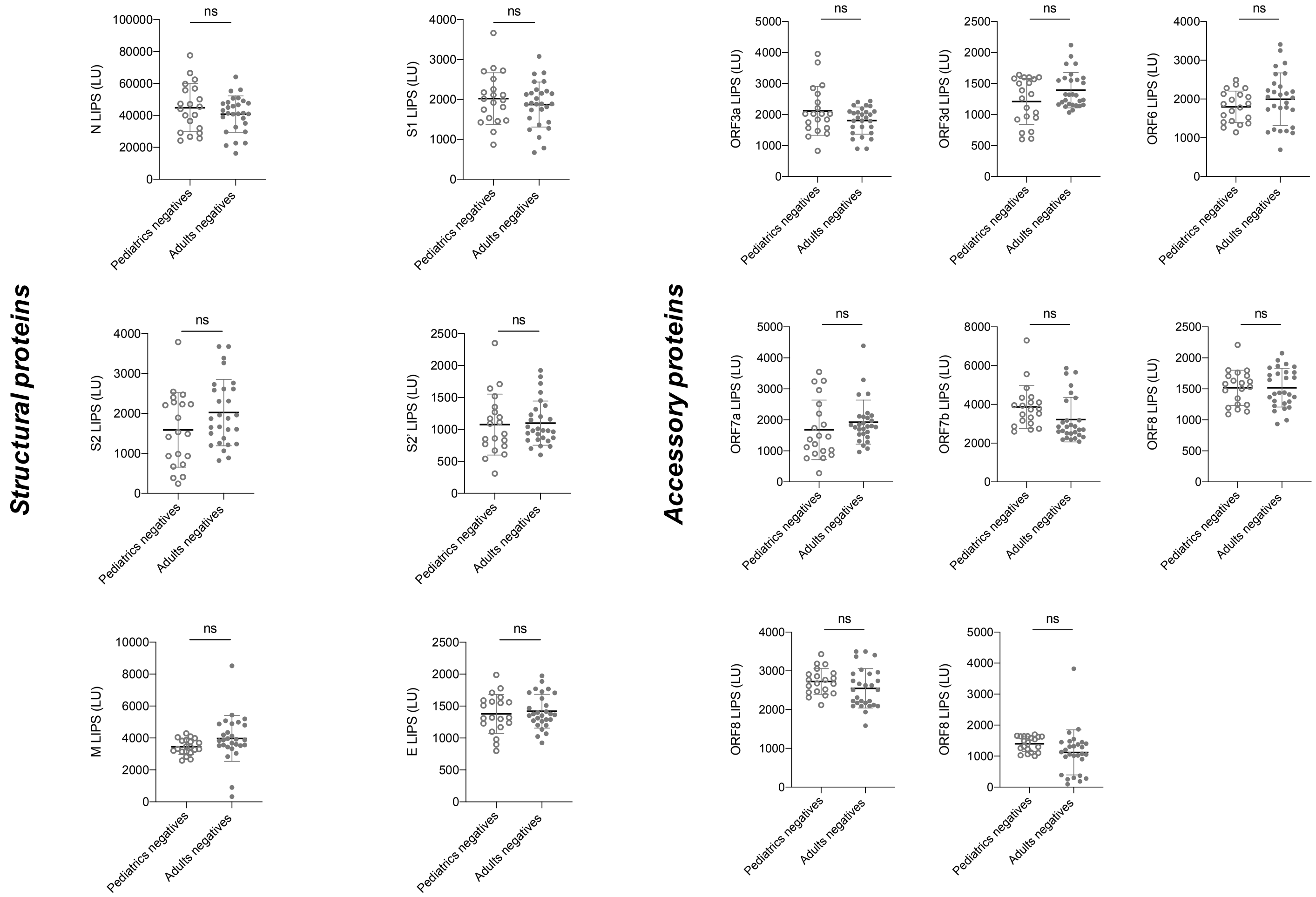

Figure 1 for reviewers only.
Comparison of antibody responses by LIPS to SARS-CoV-2 14 structural and accessory proteins in pediatric (n=20) and adult (n=28) negative pre-pandemic controls.

R1.16 - The plots compare levels of negative controls to pandemic samples that in many cases are below the pre-pandemic concentration median, therefore are negative; then, what is the meaning of comparing negative to negative? The analysis should be based on existing antibody responses (i.e. positive).
A1.16 - Antibody responses in the COVID-19 adult population are elevated for: S1, S2', N, NSP1, 3a, 3d, 7a, 7b, 8, and are negative, i.e. not elevated compared to negative pre pandemic controls for the antigens: S2, M, E, ORF6, 10. However, whilst adults may not make a specific response to ORF6, ORF10 and E antigens, differences were then seen for ORF6 and ORF10 responses when assessed for symptoms (Figure 4b), and E for adults versus children (Figure 1f and 4a). Therefore, we have not excluded negative antigens from our analysis but compared responses under different conditions. These intriguing results also give us insight into viral replication and the turnover within infected cells and cross-reactivity for previous common cold viruses for adults and children to lead to differences in immunogenicity.
We have clarified this aspect in the text in the Methods section (page 24, line 8):
"Responses were considered negative when they were not significantly elevated compared to negative pre-pandemic controls."

R1.17 - If there is concern that cutoff would vary in children vs adults due to different background (e.g. cross reactivity with CCoVs) then it should be stratified and with higher numbers. This is usually more of a concern with N and S2 than to S1. Actually, adult and children negative plasmas could be marked in different symbols in the graphs to see if they overlap
A1.17- To assess whether there was any difference in background we have compared both adult (n=28) and children (n=20) pre-pandemic controls and found no significant difference between the two populations (Reply figure 1 above). Statistical analysis was performed by student t-test and did not find any significant difference between these two pre-pandemic populations. Furthermore, we saw marked differences between infected children and controls for S1 and N, however S2 remained non-immunogenic (Figure 1).

DISCUSSION
R1.18 - As mentioned above, the main strength is the potential of the markers to distinguish adults from children serologically, in principle for diagnostics purpose and seroprevalence studies, but this is not well linked to protection, therefore the title need to be changed
A1.18 The title has been changed to:
"Accessory proteins of SARS-CoV-2 reveal distinct serological signatures in COVID-19 children"

R1.19 - There are some references that are lacking on large antibody-based studies to understand the different dynamics of SARS-CoV-2 transmission in children vs adults, including detection in plasma/serum surveys in different countries, and also in saliva, using S and N proteins. Please review and update
A1.19 – We apologize for this negligence due to the rapidly progressing COVID-19 literature. We have updated our introduction to cover further studies in this area, including the studies below:

1. Children develop robust and sustained cross-reactive spike-specific immune responses to SARS-CoV-2 infection. https://doi.org/10.1038/s41590-021-01089-8
2. Persisting Salivary IgG Against SARS-CoV-2 at 9 Months After Mild COVID-19: A Complementary Approach to Population Surveys. doi: 10.1093/infdis/jiab256.

3. COVID-19 Serology at Population Scale: SARS-CoV-2-Specific Antibody Responses in Saliva. doi: 10.1128/JCM.02204-20.
4. Antibody conversion rates to SARS-CoV-2 in saliva from children attending summer schools in Barcelona, Spain. doi: 10.1186/s12916-021-02184-1

R1.20 - This is probably one of the more relevant features that needs to be more highlighted: "the interest in antibodies to SARS-CoV-2 internal proteins will grow with the rollout of sub-unit Spike only vaccines, in order to allow the distinction between SARS-CoV-2 past infection and vaccination in specific populations and to create an estimated date of exposure given the unique rate of waning of different specificities". Furthermore, when there are evidences that antibodies to N may not be as useful for this purpose (reference lacking for this).

A1.20 – We apologize for missing out a reference on a major serological limitation in detecting infection in vaccinated individuals. Indeed, for serological markers of SARS-CoV-2 infection; anti-nucleocapsid antibody positivity may not be the ideal marker of natural infection in vaccinated individuals (https://doi.org/10.1016/j.jinf.2021.08.012), which is further supported by comparison of the response between adults and children (Weisberg et al). These have been added it to the new version of the manuscript in the Discussion section (page 20, line 18).

We also added an extra data panel as Supplemental Fig. 1f showing that serology to the accessory protein ORF8 can be used to distinguish vaccinated and infected adults samples, and the following sections:

Results (page 8, line 2):
In terms of performance as a diagnostic test, ORF8 antibodies by LIPS allows for the detection of nearly all the pediatric population with a sensitivity of 99.2% and specificity of 100% (Fig. 2g). These results were then confirmed in an in-house ELISA assay for IgG binding using recombinant proteins in both adult and paediatric plasma samples, where ORF8-protein[35] binding antibodies showed 79% sensitivity and 98.4% specificity (Supplemental Fig. 1c), whereas N and Spike-protein binding antibodies showed a sensitivity of 88% and 11% and a specificity of 97% and 99% respectively (Supplemental Fig. 1de). Furthermore, ORF8 remains a specific diagnostic tool for SARS-CoV-2 infection in vaccinated conditions. ORF8 specific IgG was assessed following vaccination with whole inactivated virion Coronavac and Spike mRNA lipoprotein BNT162b2 (Supplemental Fig. 1f), showing it is likely not incorporated within the virion.

Discussion (page 20, line 18):
“and in our hands ORF8 could distinguish between vaccinated (both mRNA and inactivated virus vaccines) and positive controls (Supplemental Fig. 1f).”

Reviewer #2 (Remarks to the Author):

Comments

COVID-19 is still unfolding. It is known that after infection the severity and morbidity is significantly different between children and adult. However, the underlying mechanism and the difference of global antibody responses are largely unknown. In this study, the authors compared the antibody responses against 14 SARS-CoV-2 proteins, also analyzed a panel of immune priming and inflammation related cytokines. They observed differences of the antibody responses and cytokine levels and also purposed the possible applications of these observed differences. This is basically an observational study, the findings may broaden our understanding of the immune response against COVID-19, especially for Children.

Major concerns:

R2.1 - This study is basically observational. To make their conclusions more convincing, the findings need to be validated at least with a small cohort of independent samples, better using an approach other than LIPS.

A2.1 – When we initially established the LIPS technique for our study we compared results to protein based ELISA, with strong correlation and consistency with results for S and N proteins (Hachim and Kavian et al., Nat Immunol 2020). The results have been consistent across 4 cohorts, and multiple assay runs in repeat. We have now added data for ELISA based protein binding IgG for N, S, and ORF8 as a supplemental figure (Supplemental figure 1 c-f) with vaccination also being addressed for both mRNA (BNT162b2, BioNTech) and inactivated virus (Coronavac, SinoVac) vaccinated patients.

We have added the following sentences:
in the Results section (page 8, line 2):
In terms of performance as a diagnostic test, ORF8 antibodies by LIPS allows for the detection of nearly all the pediatric population with a sensitivity of 99.2% and specificity of 100% (Fig. 2g). These results were then confirmed in an in-house ELISA assay for IgG binding in both adult and paediatric plasma samples, where ORF8-specific antibodies showed 79% sensitivity and 98.4% specificity (Supplemental Fig. 1c), whereas N and Spike specific antibodies showed a sensitivity of 88% and 11% and a specificity of 97% and 99% respectively (Supplemental Fig. 1de). Furthermore, ORF8 remains a specific diagnostic tool for SARS-CoV-2 infection in vaccinated conditions. ORF8 specific IgG was assessed following vaccination with whole inactivated virion Coronavac and Spike mRNA lipoprotein BNT162b2 (Supplemental Fig. 1f), showing it is likely not incorporated within the virion.

- And in the Discussion section:

"and in our hands ORF8 could distinguish between vaccinated (both mRNA and inactivated virus vaccines) and positive controls (Supplemental Fig. 1f)."

R2.2- There are a variety of publications about the COVID-19 related antibody responses, for both the structural and non-structural proteins, for example, the protein microarray related studies. These studies should be properly cited, a comprehensive comparison should be performed between this study and the previous related studies.

A2.2 – We apologize for these lacking references and have updated the Discussion section accordingly. The Discussion section now refers to further 2 microarray studies:

Page 21 line 5 for Jiang *et al*.: "Furthermore, the detection of various isotypes of antibodies for internal proteins such as IgM and IgA could also be relevant to assess the class switching, kinetic and Fc-related functions of the responses to non-surface proteins. SARS-CoV-2 proteome microarray enabled the detection of IgG and IgM to a wide-range of viral proteins, and identified alternative proteins NSP5 and ORF9b as significant antibody targets[26]."

Page 17 line 17: "Shrock *et al*., used VirScan, a DNA bacteriphage microarray, to investigate cross-reativity between SARS-CoV-2 and CCoV in COVID-19 patients[25]. They identified cross-reactive epitopes and found that cross-reactivity was weaker in severe patients than in mild patients, but samples from children were not included in this study."

R2.3 Longitudinal samples were collected. It's better to perform some longitudinal analysis for these samples.

A2.3 - Our study includes 142 longitudinal samples from 58 patients, which are necessary to assess kinetics of antibody responses. The longitudinal analysis for these samples is detailed in figure 6, including 6a: amount of blood draws, 6b: Sample collection time-line, and 6c: linear trends for LIPS values.

Minor concerns:

R2.4 - What is "LIPS"? The full name and some explanation is needed for the first appearance.

A2.4- The methods have been updated in the Methods section (Page 22-23, as below) to further describe our approach and the LIPS abbreviation now stated from the onset in the abstract and in the abbreviations list. The Luciferase Immuno Precipitation System (LIPS) is a pull down assay using immune serum from patients and transfected cell lysates to quantify antigen-specific antibody responses.

Methods (page 22, line 23):

Luciferase Immunoprecipitation System (LIPS) assays

The LIPS assay is a liquid phase immune-assay allowing the unbiased quantification of antibodies by measuring luminescence emitted by the reporter enzyme Renilla luciferase (Ruc) fused to an antigen of interest, expressed by the pRen2 vector in mammalian cells. Luciferase, a light-producing enzyme is used as a reporter. The construction of *Renilla* luciferase (Ruc) chimeric genes involves the mammalian expression vectors pREN2 in which the antigen of interest is fused in-frame with Ruc. Protein A/G beads capture the immunoglobulin-antigen complexes. Protein A/G binds predominantly to the four isotypes of IgG but can also bind, to a much lesser extent, to IgA and IgM. Therefore, our data primarily represents plasma IgG. The use of LIPS in the diagnostic of SARS-CoV-2 infection has been published by us and others (for the 14 antigens tested in the present study [24], for Spike and Nucleocapsid [61-63]).

Based on previous studies describing the structure of the SARS-CoV-2 genome[48,64], an extensive panel of 14 proteins (S1, S2, S2', E, M, N, NSP1, ORF3a, 3, 6, 7a, 7b, 8, 10) was chosen for antibody testing by LIPS. Primers for the amplification of SARS-CoV-2 proteins were designed (See protein ID and primers sequences in Supplemental table 1). Primers and cloning for the amplification of SARS-CoV-2 proteins were as previously described[24]. Constructs with pREN2-Renilla luciferase plasmid containing the SARS-CoV-2 antigen of interest were transfected into Cos1 cells using Fugene 6 (Promega) as per manufacturer's

instructions. Cells were harvested 48 hours later, lysed and sonicated, and (Ruc)-antigen yields were measured using a Luminometer plate reader (PerkinElmer) according to the protocol of Burbelo *et al.* [59] as previously described, with the following modifications [24]. Briefly, (Ruc)-antigen (at an equal concentration for each antigen at 10^7 per well) and plasma (heat inactivated and diluted 1:100) were incubated for 2 hours with shaking at 800rpm. Ultralink protein A/G beads (Thermo-Fisher) were added to the (Ruc)-antigen and serum mixture in a 96-deep-well polypropylene microtiter plate and incubated for 2 hours with shaking at 800rpm. The entire volume was then transferred into HTS plates (Millipore) and washed as previously described. The plate was read using QUANTI-Luc Gold substrate (Invivogen) as per manufacturer's instructions on a Wallac MicroBeta JET luminometer 1450 LSC & Luminescence counter and its software for analysis (PerkinElmer). Experimental controls include no plasma blank wells with (Ruc)-antigens and negative control serum from healthy donor plasmas collected prior to the COVID-19 pandemic (Table 1). The background corresponds to the LU signal from each Ruc-fusion antigen with protein A/G and substrate with no plasma. Responses were considered negative when they were not significantly elevated compared to negative pre-pandemic controls.

R2.5 - It is not correct to state "There is a lack of information on the SARS-CoV-2 antibody responses in adults or children to the virus accessory proteins which can modulate immune responses", it is not the case at least for adults.
A2.5 - We apologize for this mistaken statement and have corrected this sentence (Introduction, paragraph 3):
"Some studies have brought data on the SARS-CoV-2 antibody responses to the virus accessory proteins in the adult population (Science. 2020;370(6520), Nat Commun. 2020;11(1):3581, Nat Immunol. 21, 1293-1301 (2020)) but these data are lacking for the children population."

R2.6 - "whereas no difference was observed for S2 antibodies", please explain this.
A2.6 – In both this study and our previous study, the uncleaved form of the S2 subunit is not immunogenic, i.e. upon infection there is no increase in antibody responses above background binding of pre-pandemic controls.
There is a significant antibody response for S1 and S2' detected in COVID-19 adults compared to negative pre-pandemic control samples, and these S1 and S2' responses were markedly lower in infected children compared to infected adults. On the other hand, S2 did not trigger any antibody response in either COVID-19 adults, nor in COVID-19 children. Therefore, it appears that S2 and S2' have different antigenicity, and their conformation of the Spike is important for antibody binding.
The maturation of the viral particles by the cleavage of S2 during virus endocytosis to form the S2' fusion peptide may result in this difference in antigenicity of the 2 Spike isoforms.
We modified the sentence in the Results section (page 6, line 17) as follows:
"The levels of the two Spike antibodies, S1 and S2' were markedly lower in children compared to the adult cohort (both $p<0.0001$, Fig. 1A and C), whereas no difference was observed for S2 antibodies, revealing different antigenicity for the two Spike isoforms S2 and S2' (Fig. 1B)".

R2.7 - "we excluded the early time-point samples (<14 days of infection) from the adult cohort", what is rationale for this?
A2.7 In an effort to avoid bias in our initial results, which did not included early time points samples, we added the figure for transparency. Since then, we have been able to add 46 early timepoints (<d7) adults to the cohort with varying associated severity

(asymptomatic/mild/severe).
We apologize to the reviewer for the outdated figure and thank him for pointing out our oversight.

Reviewers' Comments:

Reviewer #1:
Remarks to the Author:

Thanks to authors for the thorough review and responses to all points. There is only one remaining itm about R1.20:
When I said that "Furthermore, when there are evidences that antibodies to N may not be as useful for this purpose (reference lacking for this)."
Authors responded adding a reference that is a commentary or a letter, but I refered to research articles that cautioned about the use of N as the sole marker of breakthrough due to potential cross.reactivity with S upon vaccination, which is not included in bibliography.
"A1.20 – We apologize for missing out a reference...."

Reviewer #2:
Remarks to the Author:
Most of the concerns were satisfied addressed except two remaining points:

1. The analysis of the longitudinal samples (R2.3). It is known that there are big person-to-person variations of antibody responses, more details and clearer trends may could be observed by analyzing the longitudinal samples of each person.
2. The S2 antibody response (R2.6). According to a variety of studies (using different platforms), it is clear that S2 can trigger significant antibody responses, though may not be as high as that of S1. However, as shown in Fig. 1b, in comparison to the negative controls, there is no S2 antibody responses to both COVID-19 adults and children. Can the author give some explanations on this? Because of the lower sensitivity of the LIPS as compared to other platforms?

REVIEWER COMMENTS

Reviewer #1 (Remarks to the Author):

Thanks to authors for the thorough review and responses to all points. There is only one remaining item about R1.20:

R1.1 When I said that "Furthermore, when there are evidences that antibodies to N may not be as useful for this purpose (reference lacking for this)." Authors responded adding a reference that is a commentary or a letter, but I referred to research articles that cautioned about the use of N as the sole marker of breakthrough due to potential cross-reactivity with S upon vaccination, which is not included in bibliography.

"A1.20 – We apologize for missing out a reference...."

A1.1. We apologise for our oversight of including a commentary on the use of N-antibodies, and have now expanded our discussion of the limitations of N-based serology in vaccinated individuals (reference of commentary on the use of N[18], and primary paper of commercial S and N tests[61]), especially as whole virion vaccines are in widespread use (commentary[59]) and N is an immundominant target (our vaccine comparison paper[60]).

The discussion section updated in yellow
"It Is possible that the interest in antibodies to SARS-CoV-2 internal proteins will grow with the rollout of sub-unit Spike only and whole inactivated virion vaccines, in order to allow the distinction between SARS-CoV-2 past infection and vaccination in specific populations and to create an estimated date of exposure given the unique rate of waning of different specificities. Inactivated vaccines represent the greatest proportion of COVID-19 vaccine doses [59], and N remains an immunodominant antibody response in CoronaVac whole inactivated virus vaccinated individuals[60]. So far, S and N based serology have been inadequate for this [18,61], and in our hands ORF8 could distinguish between vaccinated (both Spike mRNA and whole inactivated virus vaccines) and infected convalescent controls (Supplemental Fig. 1f)."

Reviewer #2 (Remarks to the Author):

Most of the concerns were satisfied addressed except two remaining points:

R2.1. The analysis of the longitudinal samples (R2.3). It is known that there are big person-to-person variations of antibody responses, more details and clearer trends may could be observed by analyzing the longitudinal samples of each person.

A2.1 We appreciate the reviewers concern over individual heterogeneity, and initially presented this data as coupled between donors. The figures of longitudinal samples (Fig. 6c and Supp Fig. 4) with paired data have been revised to show individual trends and the group average where significant, as below:

c

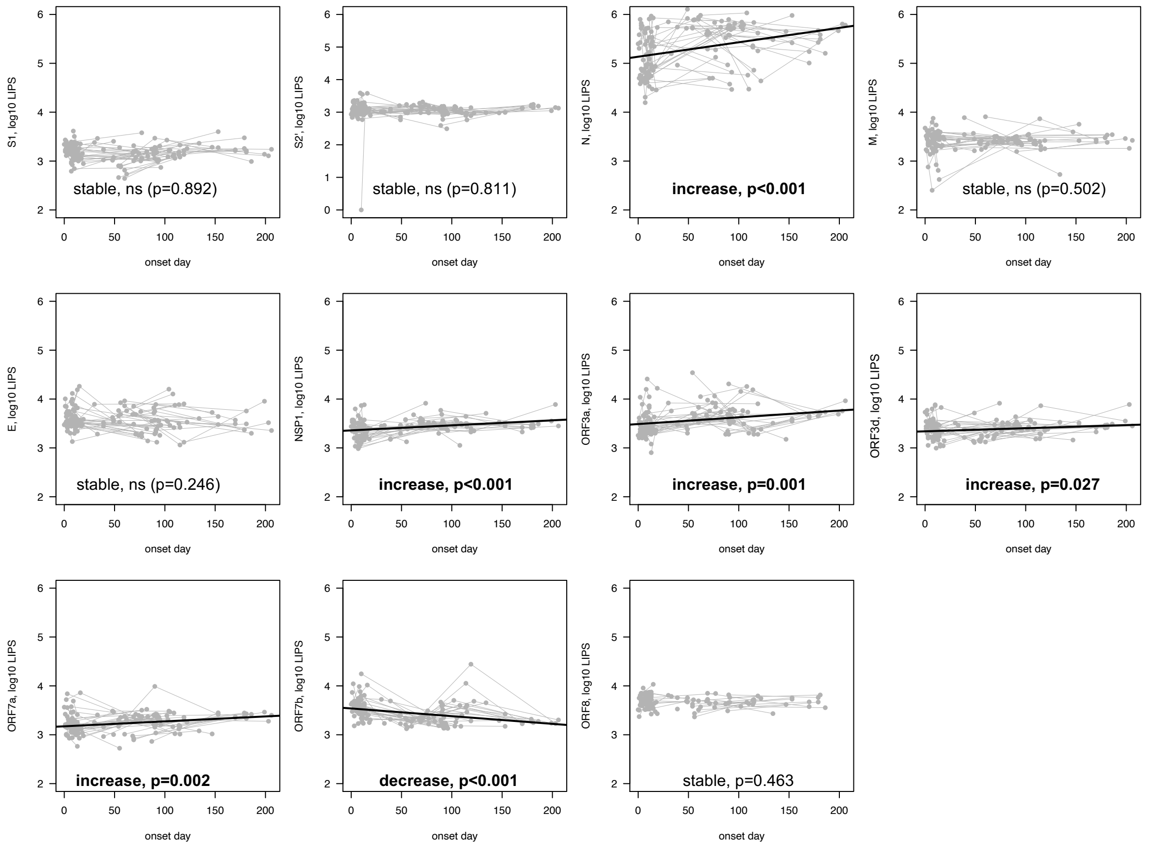

R2.2. The S2 antibody response (R2.6). According to a variety of studies (using different platforms), it is clear that S2 can trigger significant antibody responses, though may not be as high as that of S1. However, as shown in Fig. 1b, in comparison to the negative controls, there is no S2 antibody responses to both COVID-19 adults and children. Can the author give some explanations on this? Because of the lower sensitivity of the LIPS as compared to other platforms?

A2.2: To clarify, S2' is the cleaved form of the S2 and elicits a significant antibody response in adults compared to negative controls whilst S2 does not, therefore the conformation of the S2 domains as uncleaved (S2, Fig. 1b) and cleaved (S2', Fig. 1c) impacted the sensitivity of the antibody responses detected by LIPS. Therefore, we agree the S2 domain of the Spike is immunogenic, but the conformation impacts antibody binding.

The S2 and S2' responses did show some age, symptom and time dependent trends which may reflect virion formation and the extent of virus replication. Children did not mount a substantial S2' or S2 response compared to negative controls (Fig 1. bc). The S2' pediatirc response was significantly lower than adults overall (Fig. 1c) and when stratified by symptoms (Fig. 4a), and the S2 response in children was increased after day 14 of infection (Fig. 5a) which was driven by symptomatic pediatrics (Fig. 5c).

LIPS uses crude conformational antigenic targets from transfected cell lysates, whilst other platforms and studies have typically used microarray or recombinant proteins derived from various expression systems, therefore the S2 conformation and cleavage of the S2 to S2' may impact results.

Results, page 6:
"The levels of the two Spike antibodies, S1 and S2' were markedly lower in children compared to the adult cohort (both $p<0.0001$, Fig. 1a and c), whereas no difference was observed for S2 antibodies, revealing different antigenicity for the two Spike isoforms S2 and S2' (Fig. 1b)[33]."

And the S2/S2' response is further described with time and symptoms on page 14 of results.
"We previously observed that the SARS-CoV-2 antibody responses can vary in magnitude and specificity in adults between acute and convalescent to memory time-points[24]. To study the effect of time on the pediatric SARS-CoV-2 antibody specificity, we stratified pediatric responses of all 254 samples (Fig. 1 and 2) by early (<d14) versus later (≥d14) time-points of infection (Fig. 5). S2, N and ORF7a specific antibodies were significantly increased after day 14 post symptom onset. In contrast, ORF3d and ORF7b antibodies elicited a higher antibody response prior to day 14 (Fig. 5b). Finally, responses to structural proteins S1, S2', M and E and accessory proteins NSP1, ORF3a, ORF6 and ORF8 were comparable before and after day 14 (Fig. 5ab). Further stratification of samples according to time-point of sampling and symptoms was then performed (Fig. 5c) and showed significant differences for symptomatic pediatric patients for convalescent samples for key antigens, S2, ORF3d, ORF7a and ORF7b."
